# What Influence Could the Acceptance of Visitors Cause on the Epidemic Dynamics of a Reinfectious Disease?: A Mathematical Model

**DOI:** 10.1007/s10441-024-09478-w

**Published:** 2024-02-25

**Authors:** Ying Xie, Ishfaq Ahmad, ThankGod I. S. Ikpe, Elza F. Sofia, Hiromi Seno

**Affiliations:** Department of Mathematical and Information Sciences, Graduate School of Information Sciences, Tohoku University, Aramaki-Aza-Aoba 6-3-09, Aoba-ku, Sendai, 980-8579 Miyagi Japan

**Keywords:** Epidemic dynamics, Mathematical model, Ordinary differential equations, Public health, Reinfection, 92B99, 92D30, 92D25, 91D99, 00A71

## Abstract

The globalization in business and tourism becomes crucial more and more for the economical sustainability of local communities. In the presence of an epidemic outbreak, there must be such a decision on the policy by the host community as whether to accept visitors or not, the number of acceptable visitors, or the condition for acceptable visitors. Making use of an SIRI type of mathematical model, we consider the influence of visitors on the spread of a reinfectious disease in a community, especially assuming that a certain proportion of accepted visitors are immune. The reinfectivity of disease here means that the immunity gained by either vaccination or recovery is imperfect. With the mathematical results obtained by our analysis on the model for such an epidemic dynamics of resident and visitor populations, we find that the acceptance of visitors could have a significant influence on the disease’s endemicity in the community, either suppressive or supportive.

## Introduction

As the world becomes more of a global village with advances in technology and easier accessibility to different places, it is very crucial to consider side effects like the spread of diseases (Cossar 1994). The history of man is replete with stories of epidemics invading groups of people, sometimes resulting in mortality. In the long run, such diseases can disappear and recur in the future or become less deadly due to people getting immune. Some notable epidemics in history include the “Spanish” flu (1918–1919), the Black Deaths (1346–1350) which invaded Europe from Asia and recurred for three decades afterwards before getting eliminated (Brauer 2017), the SARS beginning with some infection on an airplane in 2003 (Wang and Wu 2018), and today’s pandemic situation with the COVID-19 since mid-December 2019 after the outbreak in China (CDC 2022; ECDC 2022; NIID 2022; WHO 2022). The exponentially increasing number of cases and large-scale spread of the emerging virus about the COVID-19 are being initiated and promoted by the human mobility in global and local scales (Walters et al. 2018; Du Toit 2020; Liu and Saif 2020; Munster et al. 2020; Phan et al. 2020; Ramaswamy et al. 2021; Zhang et al. 2022).

There have been many investigations concerning the effect of a people’s displacement due to social and political unrest as well as the natural migration of disease vectors to new areas on the epidemic outbreak, and especially conducted have been many theoretical/mathematical studies taking into account the possibility of individuals becoming infective during transportation and contributing significantly to transport-related infection (see Wilson (2010) and references therein; especially for the SARS virus transmission, see Wang (2014)). Not only the particular transportation with a long travel, but also the human quotidian mobility as a common phase of the human activity can be considered as one of relevant factors that could cause the spread of a transmissible disease such as influenza (WHO 2018; Seno 2020). So is the case of today’s pandemic of COVID-19 in local regions of every country (CDC 2022; ECDC 2022; WHO 2022). In the work presented by Parikh et al. (2013), a synthetic population model of the Washington DC metro area was extended to include leisure and business travelers classified as transients. The final size of the epidemic among residents was found to be remarkably higher when transients were included in the simulation of a flu-like disease outbreak. In considering the emerging diseases of wildlife, Tompkins et al. (2015) show that the key drivers of such diseases are agents from domestic sources and human-assisted exposure to infectious agents from wild populations. Talking about swine fever otherwise known as hog cholera, wild boar populations are known to serve as reservoir for the disease, thereby constituting a great challenge for domestic pig farmers, veterinarians and other stakeholders (Mur et al. 2018; Postel et al. 2018).

In this paper, we shall focus on the influence of temporal visitors in a community according to the endemicity of a transmissible disease spreading over the community. Since the globalization in business and tourism becomes crucial more and more for the economical sustainability of local communities, the condition about the acceptance of visitors would be an important part of the community’s policy for the public health about a spreading transmissible disease in and out of it. There must be such a decision on the policy by the host community as whether to accept visitors or not, the number of acceptable visitors, and the condition for acceptable visitors. Actually the importance of such a policy on the tourism regulation has been recognized more and more in the post-COVID-19 period (Rastegar et al. 2021; Volgger et al. 2021; Yan et al. 2021; Jones 2022; Okafor and Yan 2022). Making use of a mathematical model modifying the basic SIR model incorporating the regulated acceptance of an amount of temporal visitors in the community, which may be called an SIRI type of mathematical model, we shall try to consider the influence of visitors on the spread of a reinfectious disease in a community, especially assuming that a certain proportion of visitors are immune at the entry into the community.

The reinfectivity of disease in this paper means that the immunity gained by either vaccination or recovery is imperfect. For a spreading transmissible disease accompanied with a reinfectivity, the acceptance of visitors must influence the endemicity of such a disease in the community. Then the community’s policy must take account of the reinfection risk for both of residents and visitors. Actually there are transmissible diseases with a reinfectivity, including influenza (Davies et al. 1984; Hay et al. 2001; Earn et al. 2002; Price et al. 2022; Wang et al. 2022), pertussis (Hethcote 1999; van Boven et al. 2000), Lyme disease (Nadelman et al. 2012), hand, foot and mouth disease (Zhang et al. 2019), malaria (Arias et al. 2022; Rehman et al. 2022), tuberculosis (Vynnycky and Fine 1997; Horsburgh et al. 2022; Qiu et al. 2022), Ebola virus disease (MacIntyre and Chughtai 2016; Agusto 2017), chronic lung diseases (Yum et al. 2014), invasive pneumococcal disease (Lipsitch 1997), meningococcal disease (Gupta and Maiden 2001), and COVID-19 (Crawford 2022; Kumar et al. 2020; Le Page 2022; Mensah et al. 2022; Nguyen et al. 2022; Ren et al. 2022; Saad-Roy et al. 2022; Salzer et al. 2022; Shaheen et al. 2022), although the reinfectivity has been still requiring scientific researches to understand its kinetics and other nature.

According to Chowell et al. (2016), it is crucial to formulate reliable models that embody the basic transmission characteristics of specific pathogens and social scenarios. They further stated that improved models are required to capture the variation in early growth dynamics of real epidemics in order to gain better understanding of the dynamics as they reviewed trends in modeling and classifying early epidemic progression. Recently the mathematical epidemic dynamics models are being used to estimate or evaluate some epidemiological parameters and to predict the temporal variation in the morbidity about a spreading disease, making use of epidemiological data (Siettos and Russo 2013). So are particularly those on the COVID-19 spread (for example, Athayde and Alencar 2022; Kobayashi et al. 2020; Lin et al. 2022; Musa et al. 2022 ), however, this is not the case in our paper.

We are going to try to shed a light on the theoretical side about the influence of temporal visitors on the epidemic dynamics with a transmissible disease with a reinfectivity, since the acceptance of visitors under such an epidemic dynamics with the risk of reinfection could be a crucial factor to influence the endemicity in the community which must take a policy about the acceptance (Dansu and Seno 2019; Crawford 2022; Salzer et al. 2022). We shall consider one of the simplest epidemic dynamics models for a transmissible disease with reinfectivity, which is an SIRI type of mathematical model, taking into account the influence of temporal visitors accepted by a community. With the mathematical results obtained by our analysis on the model, it will be implied that the acceptance of visitors could have a significant influence on the disease’s endemicity in the community, either suppressive or supportive, depending on the risk of reinfection and the nature of accepted visitors.

## Assumptions

We consider the spread of a transmissible disease during a short-term period, that is, a season after the community starts to accept visitors from the outside, satisfying the following assumptions on the epidemic dynamics:

1. The demographic change in the resident population is negligible in the season.
2. The fatality of disease on the resident and visitor populations is negligible in the season.
3. The community starts the acceptance of a number of temporal visitors from the outside in the season after a transmissible disease has already invaded in it.
4. The entry flow of visitors is constant, that is, the net entry rate is constant independently of time.
5. The exit of visitors from the community follows a constant per capita exit rate.
6. No infected visitor is accepted by the community (i.e., the perfect quarantine), so that every accepted visitor is susceptible or immune to the disease at the entry into the community.
7. A given proportion of visitors is immune at the entry into the community.
8. Only the susceptible residents can get the vaccination to become immune, and it is not available for any visitor staying in the community.
9. Immune visitor has a possibility to get reinfected (i.e., the *imperfect* or *partial* immunity) during its stay in the community, the same as the immune resident does.
10. Infected visitor has the same exit rate as the susceptible visitor, that is, we neglect any influence of the infection on the visitor's stay in the community.

Assumption H1 indicates a time-independent constant size of resident population during the season in which the epidemic dynamics is going on. We then ignore the death due to the transmissible disease under consideration in the epidemic dynamics too, as indicated by the assumption H2. Assumption H3 indicates that the community accepts the visitors, even undergoing the spread of a transmissible disease, since the fatality of the disease is negligible with the assumption H2. No disease invasion with the visitors is assumed, as indicated by the assumption H6. From the assumptions H4 and H6, the community carries out the perfect regulation for the visitors at the entry  concerning the entry number and the quarantine. Assumption H5 mathematically means that the exit of a visitor from the community follows the homogeneous Poisson process. In a model with ordinary differential equations, it can be introduced with a constant exit rate per visitor. Assumption H7 is to reflect the situation of public health out of the community, applying the mean-field approximation for the proportion of immune visitors at the entry.

Since we assume that the community undergoes the disease spread, the assumption H8 gives the existence of a vaccination program for the residents, while it is not applied to the visitors. However, since the disease is reinfectious as assumed by the assumption H9, the immunity obtained by the vaccination or the recovery from  the disease works only to reduce the risk of reinfection. Hence the state transition in terms of the disease follows the susceptible–infective–recovered/immunized–infective (SIRI) structure in our modeling, as used for example in Gomes et al. (2004, 2005), Gökaydin et al. (2007), Stollenwerk et al. (2007), Martins et al. (2009), Pinto et al. (2010), Song et al. (2011), Georgescu and Zhang (2013), Guo et al. (2014), Pagliara et al. (2018), Buonomo (2020), Ghosh et al. (2020), Wang (2021), Srivastava et al. (2022).

Remark that the assumed reinfection is not caused by the waning or loss of immunity, which must take a certain period after getting it by the infection or vaccination. As already mentioned in the introduction section, we assume instead the imperfectness of immunity obtained by the infection or vaccination. Hence we do not introduce any specific period or time scale to get reinfected after getting the immunity. Since the infection or vaccination must generate an immunity against the disease, the assumption H9 indicates that the immunity is imperfect or partial against the infection, for example, due to the multiplicity of pathogen types (e.g., mutated variants) (Gökaydin et al. 2007; Wang et al. 2022). Because the cross-immunity is well-known for such similar pathogens by the antigen for a type of pathogen, the reinfection may be suppressed or fail to induce  an effective symptom to reproduce and discharge the pathogen to the environment.

For a simplification, the assumption H10 indicates that the exit of visitor is independent of whether the visitor is infected or not during the stay in the community. This assumption would be appropriate when the expected duration of the visitor is sufficiently shorter than the latent period, whereas it may be less appropriate when it is long. As assumed by the assumption H2, we consider a transmissible disease with little serious symptom, so that the assumption H10 would be applicable for visitors infected by such a disease.

## Model

### Generic model

**Fig. 1:**
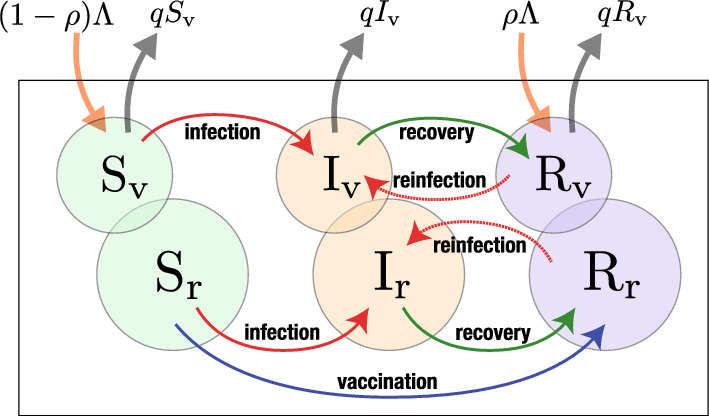
Scheme of the model for the epidemic dynamics in a community accepting temporal visitors, given by the system of (1) and (2)

With these assumptions given in the previous section, we shall consider the following model of ordinary differential equations (Fig. 1):

1 $$\begin{aligned}&\text{ Dynamics } \text{ for } \text{ the } \text{ visitor } \text{ population: } \nonumber \\&\quad \quad \left\{ \begin{aligned} \dfrac{dS_{\textrm{v}}}{dt}&= (1-\rho )\Lambda -\beta \dfrac{I_{\textrm{r}}+I_{\textrm{v}}}{N+m}S_{\textrm{v}} -qS_{\textrm{v}} ; \\ \dfrac{dI_{\textrm{v}}}{dt}&= \beta \dfrac{I_{\textrm{r}}+I_{\textrm{v}}}{N+m}S_{\textrm{v}} +\epsilon \beta \dfrac{I_{\textrm{r}}+I_{\textrm{v}}}{N+m}R_{\textrm{v}} -\gamma I_{\textrm{v}} -qI_{\textrm{v}} ; \\ \dfrac{dR_{\textrm{v}}}{dt}&= \rho \Lambda +\gamma I_{\textrm{v}} -\epsilon \beta \dfrac{I_{\textrm{r}}+I_{\textrm{v}}}{N+m}R_{\textrm{v}} -qR_{\textrm{v}} ; \end{aligned} \right. \end{aligned}$$

2 $$\begin{aligned}&\text{ Dynamics } \text{ for } \text{ the } \text{ resident } \text{ population: } \nonumber \\&\quad \quad \left\{ \begin{aligned} \dfrac{dS_{\textrm{r}}}{dt}&= -\beta \dfrac{I_{\textrm{r}}+I_{\textrm{v}}}{N+m}S_{\textrm{r}} -\sigma S_{\textrm{r}} ; \\ \dfrac{dI_{\textrm{r}}}{dt}&= \beta \dfrac{I_{\textrm{r}}+I_{\textrm{v}}}{N+m}S_{\textrm{r}} +\epsilon \beta \dfrac{I_{\textrm{r}}+I_{\textrm{v}}}{N+m}R_{\textrm{r}} -\gamma I_{\textrm{r}} ; \\ \dfrac{dR_{\textrm{r}}}{dt}&= \sigma S_{\textrm{r}}+\gamma I_{\textrm{r}} -\epsilon \beta \dfrac{I_{\textrm{r}}+I_{\textrm{v}}}{N+m}R_{\textrm{r}} , \end{aligned} \right. \end{aligned}$$

where $S_{\textrm{v}}$, $I_{\textrm{v}}$, and $R_{\textrm{v}}$ are the subpopulation sizes of susceptible, infective, and immune visitors respectively. Similarly $S_{\textrm{r}}$, $I_{\textrm{r}}$, and $R_{\textrm{r}}$ are the corresponding subpopulation sizes about the residents. The population sizes of residents and visitors staying in the community are denoted by $N = S_{\textrm{r}}+I_{\textrm{r}}+R_{\textrm{r}}$ and $m = S_{\textrm{v}}+I_{\textrm{v}}+R_{\textrm{v}}$ respectively. The resident population size *N* is constant independently of time *t*, as seen from $d(S_{\textrm{r}}+I_{\textrm{r}}+R_{\textrm{r}})/dt = 0$ for any *t* by the system (2). From the assumption H3 in Sect. 2, the community starts the acceptance of visitors from the outside in the considered season after a transmissible disease has already invaded in it. The visitor population size *m* could be reasonably assumed to be less than the population size of residents *N*: $m < N$, whereas we shall not specifically assume so but consider the mathematically general case of *m* in the subsequent sections without any constraint except for $m \ge 0$. On the other hand, as given in Sect. 3.2, we will take an assumption on the visitor population size *m* accompanying with a confinement for the net entry rate of visitors $\Lambda$.

All parameters are positive. Parameter $\rho$ is the proportion of immune visitors at the entry ($0\le \rho \le 1$). Proportion $1-\rho$ of visitors is susceptible at the entry. Parameter *q* is the per capita exit rate of visitor. Thus the expected duration of a visitor’s stay in the community is given by 1/*q*.

Parameter $\epsilon \beta$ is the reinfection coefficient for immune resident and visitor, while $\beta$ is the infection coefficient for susceptible ones. Then the infection forces for the susceptible individual and the immune individual are respectively given by $\beta (I_{\textrm{r}}+I_{\textrm{v}})/({N+m})$ and by $\epsilon \beta (I_{\textrm{r}}+I_{\textrm{v}})/({N+m})$ for both resident and visitor. Remark that, in the setup for our modeling, the visitors do not form any specific subcommunity distinct from the resident population. From the assumption H3, they are temporal visitors for tourism, business etc. For this setup, we could assume that most of visitors are independent of the others. Thus, for a mathematical simplification, the influence of their movement on the epidemic dynamics is introduced in the epidemic dynamics by the mean-field approximation. Further, although the visitors’ contribution to the infection forces would be different from the residents’ one because of the difference in the mobility/behevioral pattern, the infection forces have their same contributions in our modeling here. This modeling may be regarded as an oversimplification, though we think that our model would still worth being considered to get a cue for the discussion about the influence of visitors on the epidemic dynamics within a community.

From the assumption H9 in Sect. 2, our modeling assumes that the immunity is imperfect or partial against the infection. Because of the cross-immunity by the obtained antigen, we reasonably assume that $0\le \epsilon \le 1$ in our model, so that the reinfection coefficient $\epsilon \beta$ is not beyond the coefficient for susceptible $\beta$. That is, the infection after the vaccination or recovery from the disease generally has a smaller likelihood than that for the susceptible. For the extremal case of $\epsilon =1$, the vaccination or recovery does not work at all to reduce the risk of reinfection. For $\epsilon = 0$, the recovery and vaccination give the perfect immunity so that there is no likelihood of reinfection. Thus the parameter $\epsilon$ means an index for the likelihood of reinfection after the recovery or vaccination, so that it can be regarded as an index for the risk of reinfection. Remark here again that the reinfection in our modeling is assumed to be not due to the waning of immunity (like for the SIRS models) but due to the imperfect immunity, and hence also the vaccinated individual has a risk to get infected, as introduced by the assumption H9.

Parameter $\gamma$ is the recovery rate of an infective individual, and the recovered individual gets immunity, which is however imperfect. Only the susceptible residents can get the vaccination, with rate $\sigma$, and it is not available for any visitor staying in the community. Since the vaccination is imperfect from the assumption H9, it works to reduce the risk of infection but is unable to protect the vaccinated individual from the infection.

### Assumption for the visitor population size in the community

According to the dynamics for the visitor population (1), we have

$$\begin{aligned} \dfrac{dm}{dt} = \Lambda - qm, \end{aligned}$$

where $m = m(t):=S_{\textrm{v}}(t)+I_{\textrm{v}}(t)+R_{\textrm{v}}(t)$ is the visitor population size at time *t*, $\Lambda$ the net entry rate of visitors, and *q* the per capita exit rate of visitor. Now let us consider the stationary situation with respect to the temporal change of visitor population size. This means that the number of visitors is assumed to be stationary, which may be regarded as a consequence of the regulation of their entry by the community, following the assumptions H4 and H6 given in Sect. 2. Therefore we mathematically assume the situation to satisfy that $dm/dt = 0$. Hence we put $\Lambda = qm$, and hereafter treat the visitor population size *m* as a positive constant.

### The Initial Condition

Following the assumption of the stationary visitor population size with $\Lambda = qm$ as given in Sect. 3.2, we have the following dynamics for the visitor population at the disease-free state:

$$\begin{aligned} \left\{ \begin{aligned} \dfrac{dS_{\textrm{v}}}{dt}&= (1-\rho )qm -qS_{\textrm{v}} ; \\ \dfrac{dR_{\textrm{v}}}{dt}&= \rho qm -qR_{\textrm{v}}, \end{aligned} \right. \end{aligned}$$

where $R_v$ means the subpopulation size of immune visitors in the disease-free community. It can be easily found that this dynamics results in an eventual approach to the equilibrium state such that $(S_{\textrm{v}}, R_{\textrm{v}})\rightarrow ((1-\rho )m, \rho m)$ as $t\rightarrow \infty$ for any non-negative initial condition with  $S_{\textrm{v}}(0) \ge 0$ and $R_{\textrm{v}}(0) = m -S_{\textrm{v}}(0)\ge 0$. For this reason, let us assume the following initial condition for the epidemic dynamics with the model given by the system of (1) and (2):

3 $$\begin{aligned} (S_{\textrm{v}}, I_{\textrm{v}}, R_{\textrm{v}}, S_{\textrm{r}}, I_{\textrm{r}}, R_{\textrm{r}}) = \big ((1-\rho )m, 0, \rho m, S_{\textrm{r}0}, I_{\textrm{r}0}, R_{\textrm{r}0}\big ), \end{aligned}$$

where $S_{\textrm{r}0}+ I_{\textrm{r}0}+ R_{\textrm{r}0} = N$ (a positive constant) with $S_{\textrm{r}0}>0$, $I_{\textrm{r}0}>0$ and $R_{\textrm{r}0}\ge 0$. This initial condition defines the situation when the community starts the acceptance of visitors from the outside, even under the existence of disease in it. The setup of this initial condition as our modeling follows the assumption H3 in Sect. 2.

## Basic Reproduction Number

In the biological/epidemiological context, the basic reproduction number is defined as the expected number of new cases of infection caused by an infective individual within a population consisting of susceptible contacts only (for a useful  review about the definition, the translation, and the practical application, see Delamater et al. (2019)). Such a situation can never occur for the epidemic dynamics in reality or even by any mathematical model. This is because the secondary infection itself changes the situation of population where the disease spreads. Increase of infectives must reduce the likelihood of the contact between an infective and an susceptible since the likelihood to contact the other infectives becomes less negligible. For this reason, the basic reproduction number must be mathematically defined to match the above conceptual definition in the biological/epidemiological context. Briefly saying, it is mathematically defined as the *supremum* of the expected number of new cases of infection caused by an infected individual at the stage of disease invasion in the community (Seno 2022). Following this definition in a biological/epidemiological sense, a mathematical theory can be used to derive the basic reproduction number about the mathematical model of epidemic dynamics, for example, as the spectral radius of a specific matrix, called the “next generation matrix”, for a system of ordinary differential equations governing an epidemic dynamics (see Diekmann et al. (2013) for a complete reference, or see Lewis et al. (2019) or van den Driessche (2017) for the further review). The choice of the derivation way of ${\mathscr {R}}_0$ may depend on the nature of the model. As well known on the nature of the basic reproduction number, the invasion of a transmissible disease in the community is successful if ${\mathscr {R}}_0> 1$, while it fails if ${\mathscr {R}}_0 < 1$. In this context, the invasion success means the increase of infectives in the early stage of the disease spread after a sufficiently small number of infectives appear in the community, and the invasion failure implies the decrease.

For our model given by the system of (1) and (2), we can derive the following formula of the basic reproduction number ${\mathscr {R}}_0$ (Appendix A):

4 $$\begin{aligned} {\mathscr {R}}_0&= \underbrace{ \dfrac{\, 1\, }{\gamma } }_{\begin{array}{c} {\text {the expected }}\\ {\text {duration of }}\\ {\text {infectivity.}} \end{array}} \times \ \Big [ \hspace{-5pt} \underbrace{ \beta \dfrac{N}{N+m} }_{\begin{array}{c} {\text {the supremum}} \\ {\text {of the expected}} \\ {\text {new cases per}} \\ {\text {unit time for the}} \\ {\text {resident.}} \end{array}} + \underbrace{ \Big \{ \beta \dfrac{(1-\rho )m}{N+m} +\ \epsilon \beta \dfrac{\rho m}{N+m} \Big \} }_{\begin{array}{c} {\text {the supremum of the expected new}}\\ {\text {cases per unit time for the visitor,}}\\ {\text {given by the sum of secondary }}\\ {\text {infections for susceptible}} \\ {\text {and immune visitors.}} \end{array}} \Big ] \nonumber \\&= {\mathscr {R}}_{00} \Big \{ 1- (1-\epsilon )\rho \dfrac{\mu }{1+\mu } \Big \}, \end{aligned}$$

where $\mu := m/N$, and for a convenience in the following arguments, we define ${\mathscr {R}}_{00}:=\beta /\gamma$, which is the basic reproduction number for the community when no visitor comes in (i.e., $m = 0$). Note that this basic reproduction number is fundamental for the epidemic dynamics in the community after it starts the acceptance of visitors.

From the formula (4), we can immediately find that the basic reproduction number ${\mathscr {R}}_0$ is less than 1 independently of the nature of accepted visitors if ${\mathscr {R}}_{00} < 1$. Hence, if the disease fails its invasion in the community with $\mathscr {R}_{00} < 1$ before starting the acceptance of visitors, the number of infectives in the community cannot turn to increase in the early period after the acceptance of visitors starts. As we will see in the later sections of the analysis on our model, this is valid only in the early period after the acceptance of visitors starts.

As for the dependence of ${\mathscr {R}}_0$ on the nature of accepted visitors, we note that ${\mathscr {R}}_0$ is monotonically decreasing in terms of $\mu$ when the visitors contain some immune ones (i.e., $\rho > 0$). Moreover, ${\mathscr {R}}_0$ becomes smaller as the proportion of immune visitors at the entry $\rho$ gets larger. If any visitor is susceptible, that is, when $\rho = 0$, there is no contribution of the visitors to the basic reproduction number ${\mathscr {R}}_0$, that is, ${\mathscr {R}}_0 = {\mathscr {R}}_{00}$.

As an extremal case, if the immunity gained by the vaccination or recovery from the disease does not work at all to reduce the risk of reinfection, that is, if $\epsilon = 1$, the basic reproduction number ${\mathscr {R}}_0$ is independent of the acceptance of visitors. This is easily understandable, since the reinfection is regarded as the same as the infection for the susceptible so that the immune individual is regarded as equivalent to the susceptible according to the epidemic dynamics when $\epsilon = 1$. Such an extreme case may be regarded as corresponding to an SIS type of the epidemic dynamics, where the state transition in terms of the disease follows the susceptible–infective–susceptible structure.

As the other extremal case, if the immunity is perfectly effective to make the immune individual unable to be reinfected, that is, if $\epsilon = 0$, the entry of immune visitors works to reduce the value of ${\mathscr {R}}_0$ for the community. This extremal case may be regarded as corresponding to an SIR type of the epidemic dynamics, where the state transition in terms of the disease follows the susceptible–infective–removed structure.

**Fig. 2:**
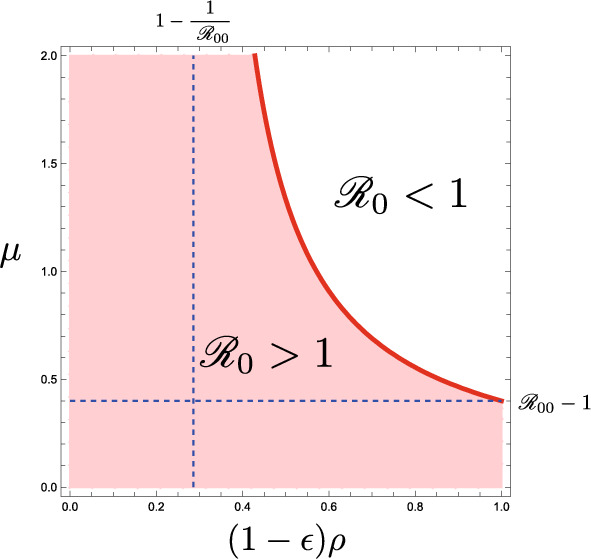
The dependence of the basic reproduction number ${\mathscr {R}}_0$ given by (4) on parameters $(1-\epsilon )\rho$ and $\mu := m/N$. Numerically drawn with ${\mathscr {R}}_{00} = 1.4$

From these arguments with the basic reproduction number $\mathscr {R}_0$ given by (4), we can get the following result on the influence of the acceptance of visitors in the early period after the community starts the acceptance of visitors (Fig. 2):

### Theorem 4.1

The acceptance of visitors influences the basic reproduction number ${\mathscr {R}}_0$ given by (4) for the epidemic dynamics with the system of (1) and (2) as follows:

(i) The acceptance of visitors makes ${\mathscr {R}}_0$ smaller than ${\mathscr {R}}_{00}$ if and only if the visitors contain some immune, and its decline becomes greater as the number of accepted visitors gets larger;
(ii) When ${\mathscr {R}}_{00} > 1$, if
  $$\begin{aligned} \rho \le \rho _{\infty }^0 :=\dfrac{1}{1-\epsilon }\Big (1-\dfrac{1}{\ {\mathscr {R}}_{00}}\Big ), \end{aligned}$$
  then ${\mathscr {R}}_0 > 1$ independently of the number of accepted visitors;
(iii) When ${\mathscr {R}}_{00} > 1$, if and only if $\rho > \rho _{\infty }^0$, the acceptance of visitors so many as
  $$\begin{aligned} \mu > \dfrac{1-1/{\mathscr {R}}_{00}}{(1-\epsilon )\rho -(1-1/{\mathscr {R}}_{00})} \end{aligned}$$
  makes $${\mathscr {R}}_0 < 1$$.

The result (*i*) in Theorem 4.1 means that the acceptance of visitors does not help the spread of a transmissible disease as long as ${\mathscr {R}}_{00} \le 1$, and instead it could work to suppress the spread if the community accepts a sufficiently large number of visitors with a sufficiently large proportion of immune, as indicated by the results (*ii*) and (*iii*).

Note that these arguments and result are about the effect of the acceptance of visitors on the temporal change of the number of infectives only in the early period after the community starts the acceptance of visitors. They cannot be necessarily applied for its later temporal change. It may be possible that the number of infectives turns to increase in a later period, independently of what final state the epidemic dynamics approaches, as we will actually see in the later sections of the analysis on our model.

## Non-Dimensional Transformation of the System

Since the population sizes of visitors and residents are assumed constant independently of time, the above six dimensional system of (1) and (2) can be mathematically reduced to the following closed four dimensional one, making use of $S_{\textrm{v}}+I_{\textrm{v}}+R_{\textrm{v}}=m$ and $S_{\textrm{r}}+I_{\textrm{r}}+R_{\textrm{r}}=N$:

$$\begin{aligned} \dfrac{dS_{\textrm{v}}}{dt}&= (1-\rho )qm -\beta \dfrac{I_{\textrm{r}}+I_{\textrm{v}}}{N+m}S_{\textrm{v}} -qS_{\textrm{v}} ; \\ \dfrac{dI_{\textrm{v}}}{dt}&= \beta \dfrac{I_{\textrm{r}}+I_{\textrm{v}}}{N+m}S_{\textrm{v}} +\epsilon \beta \dfrac{I_{\textrm{r}}+I_{\textrm{v}}}{N+m} (m-S_{\textrm{v}}-I_{\textrm{v}}) -\gamma I_{\textrm{v}} -qI_{\textrm{v}} ; \\ \dfrac{dS_{\textrm{r}}}{dt}&= -\beta \dfrac{I_{\textrm{r}}+I_{\textrm{v}}}{N+m}S_{\textrm{r}} -\sigma S_{\textrm{r}} ; \\ \dfrac{dI_{\textrm{r}}}{dt}&= \beta \dfrac{I_{\textrm{r}}+I_{\textrm{v}}}{N+m}S_{\textrm{r}} +\epsilon \beta \dfrac{I_{\textrm{r}}+I_{\textrm{v}}}{N+m} (N-S_{\textrm{r}}-I_{\textrm{r}}) -\gamma I_{\textrm{r}}. \end{aligned}$$

Now we apply the following transformation of variables and parameters for this four dimensional system:

$$\begin{aligned}&\tau : = \gamma t;\ x_{\textrm{v}}(t):=\dfrac{S_{\textrm{v}}(t)}{m};\ y_{\textrm{v}}(t):=\dfrac{I_{\textrm{v}}(t)}{m};\ x_{\textrm{r}}(t):=\dfrac{S_{\textrm{r}}(t)}{N};\ y_{\textrm{r}}(t):=\dfrac{I_{\textrm{r}}(t)}{N};\ \\&\mu :=\dfrac{\, m\, }{N};\ c:=\dfrac{\, q\, }{\gamma };\ \omega :=\dfrac{\, \sigma \, }{\gamma }, \end{aligned}$$

and then we can derive the following non-dimensinalized system:

5 $$\begin{aligned} \begin{aligned} \dfrac{dx_{\textrm{v}}}{d\tau }&= (1-\rho )c -\mathscr {R}_{00}\dfrac{y_{\textrm{r}}+\mu y_{\textrm{v}}}{1+\mu }x_{\textrm{v}} -cx_{\textrm{v}} ; \\ \dfrac{dy_{\textrm{v}}}{d\tau }&= \mathscr {R}_{00}\dfrac{y_{\textrm{r}}+\mu y_{\textrm{v}}}{1+\mu }x_{\textrm{v}} +\epsilon {\mathscr {R}}_{00}\dfrac{y_{\textrm{r}}+\mu y_{\textrm{v}}}{1+\mu } (1-x_{\textrm{v}}-y_{\textrm{v}}) - (1+c )y_{\textrm{v}} ; \\ \dfrac{dx_{\textrm{r}}}{d\tau }&= -{\mathscr {R}}_{00}\dfrac{y_{\textrm{r}}+\mu y_{\textrm{v}}}{1+\mu }x_{\textrm{r}} -\omega x_{\textrm{r}} ; \\ \dfrac{dy_{\textrm{r}}}{d\tau }&= \mathscr {R}_{00}\dfrac{y_{\textrm{r}}+\mu y_{\textrm{v}}}{1+\mu }x_{\textrm{r}} +\epsilon {\mathscr {R}}_{00}\dfrac{y_{\textrm{r}}+\mu y_{\textrm{v}}}{1+\mu } (1-x_{\textrm{r}}-y_{\textrm{r}}) - y_{\textrm{r}}, \end{aligned} \end{aligned}$$

where ${\mathscr {R}}_{00}:= \beta /\gamma$ as before. Remark that the symbol ${\mathscr {R}}_{00}$ is formally introduced now as a dimensionless parameter for the non-dimensionalized system given by (5), while its meaning is given in Sect. 4 as the basic reproduction number for the community when no visitor comes in. The initial condition (3) now becomes

$$\begin{aligned} (x_{\textrm{v}}(0), y_{\textrm{v}}(0), x_{\textrm{r}}(0), y_{\textrm{r}}(0) ) = (1-\rho , 0, x_{\textrm{r}0}, y_{\textrm{r}0}) \end{aligned}$$

with $x_{\textrm{r}0}:= S_{\textrm{r}0}/N =1-I_{\textrm{r}0}/N = 1-y_{\textrm{r}0}$, where we set *R*_r0_ = 0 for a simplicity that there is no immune resident at the initial, which will not affect any mathematical result obtained in our analysis on the model. In the following sections, we shall analyze the non-dimensionalized system (5) to investigate the nature of the epidemic dynamics by our model with the system of (1) and (2).

## Dynamics Without Reinfection

In this section, we consider the system without reinfection, that is, with $\epsilon = 0$, while we will consider our model only with $\epsilon > 0$ in the subsequent sections. For the system (5) with $\epsilon = 0$, we can easily find that $x_{\textrm{r}}\rightarrow 0$ and $y_{\textrm{r}}\rightarrow 0$ as $\tau \rightarrow \infty$. In other words, since the epidemic dynamics for the resident population is governed by an SIR model with the continuous vaccination measure for the susceptibles, the disease eventually disappears in the resident population, and the residents come to make no contribution to the epidemic dynamics. This means that, for our model of (1) and (2) *without reinfection*, no endemic state can be established as long as the community does not accept any visitor from the outside. Thus we consider the case of $\mu > 0$ hereafter in this section, when the community accepts the visitors.

By the local stability analysis with the eigenvalues of Jacobi matrix at the equilibrium, we can easily find that the endemic equilibrium $E_{+0}({{\widetilde{x}}}_{\textrm{v}}^*,\, \widetilde{y}_{\textrm{v}}^*, 0, 0)$ with

6 $$\begin{aligned} {{\widetilde{x}}}_{\textrm{v}}^* = \dfrac{(1+c)(1+\mu )}{\mathscr {R}_{00}\mu }; \quad {{\widetilde{y}}}_{\textrm{v}}^* = c\Big (\dfrac{1-\rho }{1+c}-\dfrac{1+\mu }{{\mathscr {R}}_{00}\mu }\Big ) \end{aligned}$$

is locally asymptotically stable when it exists. Then we can get the following theorem on the epidemic dynamics given by (5) with $\epsilon = 0$ (Appendix B):

### Theorem 6.1

For the system (5) with $\epsilon = 0$, if and only if the condition

7 $$\begin{aligned} {\mathscr {R}}_{00} >\dfrac{1}{1-\rho }\Big (1+\frac{\, 1\, }{\mu }\Big ) (1+c ) \end{aligned}$$

is satisfied, the endemic equilibrium $E_{+0}({{\widetilde{x}}}_{\textrm{v}}^*,\, {{\widetilde{y}}}_{\textrm{v}}^*, 0, 0)$ with (6) uniquely exists, and it is globally asymptotically stable. Otherwise, the disease-eliminated equilibrium $E_{00}(1-\rho , 0, 0, 0)$ is globally asymptotically stable.

**Fig. 3:**
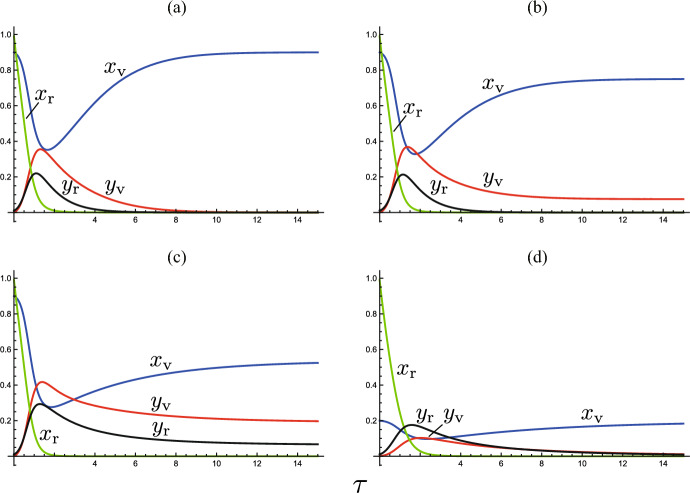
Temporal variations by the system (5). Numerically drawn with **a** $(\epsilon , \mu , \rho ) = (0.0, 0.2, 0.1)$ (${\mathscr {R}}_{0} = 7.87$); **b** $(\epsilon , \mu , \rho ) = (0.0, 0.5, 0.1)$ (${\mathscr {R}}_{0} = 7.73$); **c** $(\epsilon , \mu , \rho ) = (0.1, 0.2, 0.1)$ (${\mathscr {R}}_{0} = 7.88$); **d** $(\epsilon , \mu , \rho ) = (0.1, 0.5, 0.8)$ (${\mathscr {R}}_{0} = 6.08$); and commonly ${\mathscr {R}}_{00} = 8.0$; $c = 1.0$; $\omega = 1.0$; $(x_{\textrm{v}}(0), y_{\textrm{v}}(0), x_{\textrm{r}}(0), y_{\textrm{r}}(0) ) = (1-\rho , 0.0, 0.99, 0.01)$. In **a**, **d**, the system approaches the disease-eliminated equilibrium, and in **b**, **c**, it approaches the endemic equilibrium

See Fig. 3(a, b) for numerical examples.

From the basic reproduction number ${\mathscr {R}}_0$ given by (4) with $\epsilon = 0$, we can easily find that $\mathscr {R}_0 > 1$ if the condition (7) is satisfied, while the inverse does not necessarily hold. Hence we can get the following result:

### Corollary 6.1.1

Even when the disease successfully invades in the community with ${\mathscr {R}}_{0} > 1$, the disease without reinfection eventually gets eliminated unless the condition (7) is satisfied.

When the condition (7) is unsatisfied with ${\mathscr {R}}_{0} > 1$, the number of infectives increases at the initial stage of disease spread in the community, and then it eventually turns to decrease toward zero, as numerically exemplified in Fig. 3(a).

For $\rho = 1$, the condition (7) cannot be satisfied:

### Corollary 6.1.2

If the community accepts only immune visitors, the epidemic dynamics without reinfection necessarily approaches the disease-eliminated equilibrium.

Therefore, when the reinfection is impossible/negligible, the acceptance of visitors with a high immune proportion at the entry does not cause the endemicity of disease.

We must remark that the endemic equilibrium $E_{+0}$ is sustained only by the visitor subpopulation, while no resident contributes to the epidemic dynamics at the equilibrium because all residents have eventually become immune by the past infection or vaccination [Fig. 3(a, b)]. From Theorem 6.1, for the disease with a sufficiently high infectivity, the acceptance of many visitors with a sufficiently small immune proportion at the entry can induce  such an apparent endemicity in the community. Therefore, as seen in Fig. 3(a, b), when the community successfully controls and reduces the number of visitors to make $\mu$ sufficiently small, or if the community suspends accepting the visitors, the endemic state can be disrupted, and then the disease gets eliminated in the community. However, as we will see in the subsequent sections, if the disease is accompanied by a reinfectivity, this could not be the case [Fig. 3(c, d)].

## Dynamics with no Visitor

Next we consider the model of (1) and (2) with reinfection, that is, with $\varepsilon > 0$, when the community does not accept any visitor from the outside. Thus we analyze the following system derived from (5) with $x_{\textrm{v}} = y_{\textrm{v}}\equiv 0$ and $\mu = 0$:

8 $$\begin{aligned} \begin{aligned} \dfrac{dx_{\textrm{r}}}{d\tau }&= -{\mathscr {R}}_{00}y_{\textrm{r}}x_{\textrm{r}} -\omega x_{\textrm{r}} ; \\ \dfrac{dy_{\textrm{r}}}{d\tau }&= (1-\epsilon ){\mathscr {R}}_{00}y_{\textrm{r}}x_{\textrm{r}} -\epsilon {\mathscr {R}}_{00}y_{\textrm{r}}^2-\big (1-\epsilon {\mathscr {R}}_{00}\big )y_{\textrm{r}}. \end{aligned} \end{aligned}$$

As already mentioned in Sect. 4, the basic reproduction number for this epidemic dynamics is defined as ${\mathscr {R}}_0 = {\mathscr {R}}_{00}= \beta /\gamma$. It is easy to find that there are two feasible equilibria for this system of $(x_{\textrm{r}}, y_{\textrm{r}})$: the disease-eliminated equilibtium $E_0(0, 0)$ and the endemic equilibrium $E_+\big (0, 1-1/(\epsilon {\mathscr {R}}_{00})\big )$. The endemic equilibrium $E_{+}$ exists when and only when $\epsilon {\mathscr {R}}_{00} > 1$.

**Fig. 4:**
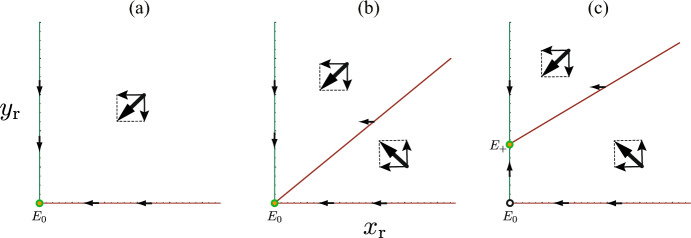
Application of the isocline method for the system with no visitor (8) when **a** $\epsilon {\mathscr {R}}_{00} < 1$; **b** $\epsilon {\mathscr {R}}_{00} = 1$; (c) $\epsilon {\mathscr {R}}_{00} > 1$

Making use of the local stability analysis for the equilibrium of the system (8), we can easily find that the equilibrium $E_0$ is locally asymptotically stable if $\epsilon {\mathscr {R}}_{00} < 1$, and unstable if $\epsilon \mathscr {R}_{00} > 1$. The endemic equilibrium $E_+$ is locally asymptotically stable whenever it exists. Taking account of the result on the local stability of equilibria, the isocline method for the two dimensional system (8) can further give the following result (see Fig. 4):

### Theorem 7.1

For the system (8) with no visitor,

(i) if and only if $\epsilon {\mathscr {R}}_{00} \le 1$, the disease-eliminated equilibrium $E_0$ is globally asymptotically stable;
(ii) if and only if $\epsilon {\mathscr {R}}_{00} > 1$, the endemic equilibrium $E_+$ exists and is globally asymptotically stable, while $E_0$ is unstable.

This result was shown for a mathematically equivalent SIRI model in Gomes et al. (2005).

From this result, we find that, even with the basic reproduction number ${\mathscr {R}}_{00} > 1$, the community approaches the disease-eliminated equilibrium $E_0$ if ${\mathscr {R}}_{00} \le 1/\epsilon$ with $0< \epsilon < 1$. In such a case, the number of infectives increases at the initial stage of disease spread in the community, and then it eventually turns to decrease toward zero. If and only if the basic reproduction number is sufficiently large as ${\mathscr {R}}_{00} > 1/\epsilon$, the disease becomes endemic in the community.

As shown in the previous section, no endemicity arises in the community with a non-reinfectious disease when no visitor is accepted. Now the result obtained in this section indicates that the endemicity of a disease can arise in the community even with no visitor if the disease has both sufficiently high infectivity (${\mathscr {R}}_{00} > 1$) and sufficiently high reinfectivity ($\epsilon >1/{\mathscr {R}}_{00}$).

## Dynamics with Visitors

### Disease-Eliminated Equilibrium

For the system (5) with visitors, we can get the following result on the local stability of the disease-eliminated equilibrium $E_{00}(1-\rho , 0, 0, 0)$ (Appendix C):

#### Theorem 8.1

The disease-eliminated equilibrium $E_{00}$ is unstable if

9 $$\begin{aligned} \epsilon {\mathscr {R}}_{00} > G(\mu , \rho ) := \left[ \Big \{ \frac{\, 1\, }{\epsilon } (1-\rho ) +\rho \Big \} \dfrac{1}{1+c} \dfrac{\mu }{1+\mu } + \dfrac{1}{1+\mu } \right] ^{-1}, \end{aligned}$$

while it is locally asymptotically stable if the inverse inequality of (9) is satisfied.

It can be easily seen that the condition (9) becomes equivalent to (7) as $\epsilon \rightarrow 0$. This shows a mathematical consistency of Theorem 8.1 to Theorem  6.1.

Moreover we can prove the following result about the relation of the basic reproduction number ${\mathscr {R}}_0$ defined by (4) to the condition (9) (Appendix D):

#### Corollary 8.1.1

When ${\mathscr {R}}_0 \le 1$, the disease-eliminated equilibrium $E_{00}$ is locally asymptotically stable.

This result is consistent with the biological/epidemiological meaning of the basic reproduction number with respect to the invasion success of a disease in a population, which was described in Sect. 4 and references therein. Under the condition that $\mathscr {R}_0 < 1$ at the initial stage of a disease invasion in a population, the number of infectives is expected to decrease toward the disease elimination. In a sense of epidemic dynamics, such a decline of the infective subpopulation toward the elimination must follow the locally asymptotic stability of the disease-free equilibrium (as referred in most literatures), which corresponds here to the disease-eliminated equilibrium $E_{00}$. The result of Corollary 8.1.1 shows this consistency.

### Endemic Equilibrium

From the equations of (5), if the endemic equilibrium $E_{++}(x_{\textrm{v}}^*, y_{\textrm{v}}^*, x_{\textrm{r}}^*, y_{\textrm{r}}^*)$ with $y_{\textrm{v}}^*>0$ and $y_{\textrm{r}}^*>0$ exists for $\rho <1$, then it must satisfy that

10 $$\begin{aligned} \begin{aligned}&{\mathscr {R}}_{00}\dfrac{y_{\textrm{r}}^*+\mu y_{\textrm{v}}^*}{1+\mu } =c\dfrac{1-\rho -x_{\textrm{v}}^*}{x_{\textrm{v}}^*}, \\&y_{\textrm{v}}^* = \dfrac{1-\rho -x_{\textrm{v}}^*}{x_{\textrm{v}}^*}\, \dfrac{(1-\epsilon )x_{\textrm{v}}^*+\epsilon }{1+1/c+\epsilon (1-\rho -x_{\textrm{v}}^*)/x_{\textrm{v}}^*}, \\&y_{\textrm{r}}^* = \dfrac{1-\rho -x_{\textrm{v}}^*}{x_{\textrm{v}}^*}\, \dfrac{\epsilon }{1/c+\epsilon (1-\rho -x_{\textrm{v}}^*)/x_{\textrm{v}}^*}, \end{aligned} \end{aligned}$$

and $x_{\textrm{r}}^* = 0$. In contrast, at the endemic equilibrium $E_{++}$ for $\rho = 1$, we have

11 $$\begin{aligned} \begin{aligned}&\epsilon {\mathscr {R}}_{00}\dfrac{y_{\textrm{r}}^*+\mu y_{\textrm{v}}^*}{1+\mu }(1-y_{\textrm{v}}^*) -(1+c)y_{\textrm{v}}^* =0, \\&\epsilon {\mathscr {R}}_{00}\dfrac{y_{\textrm{r}}^*+\mu y_{\textrm{v}}^*}{1+\mu }(1-y_{\textrm{r}}^*) -y_{\textrm{r}}^* =0, \end{aligned} \end{aligned}$$

and $x_{\textrm{v}}^* = 0$, $x_{\textrm{r}}^* = 0$, instead of (10). We can obtain the following result on the existence of $E_{++}$ (Appendix E):

#### Theorem 8.2

The endemic equilibrium $E_{++}$ uniquely exists if and only if the condition (9) is satisfied.

Hence, when the disease-eliminated equilibrium $E_{00}$ is locally asymptotically stable, the endemic equilibrium $E_{++}$ does not exist, and when $E_{00}$ is unstable, $E_{++}$ uniquely exists.

For the local stability of the endemic equilibrium $E_{++}$ for (5), we can get the following result:

#### Theorem 8.3

When the endemic equilibrium $E_{++}$ exists, it is locally asymptotically stable.

This theorem can be proved by the eigenvalue analysis on the Jacobi matrix for (5) at the endemic equilibrium $E_{++}$, applying the Routh-Hurwitz criterion (Appendix F). Although we could not get any analytical result on the global stability of the endemic equilibrium $E_{++}$, our numerical calculations imply that it is globally asymptotically stable when it exists. We then have the mathematical consistency of Theorems 8.1, 8.2, and 8.3 to Theorem  6.1 as for the case with no reinfection, $\epsilon = 0$.

## Influence of the Acceptance of Visitors

### Shift in Endemicity

As the important preliminary for our analysis on the model, we can easily find the following features of $G(\mu , \rho )$ defined in (9):

- $$G(0, \rho ) = 1$$.
- $G(\mu , \rho )$ is monotonically increasing in terms of $\rho$ for any $\mu >0$.
- $$G(\mu , \rho )$$ is $$\left\{ \begin{aligned}&\text{ monotonically } \text{ increasing } \text{ in } \text{ terms } \text{ of } \mu \ \text{ if }\ \rho > \rho _s:= 1-\dfrac{c}{1/\epsilon - 1}; \\&\text{ constant } (\equiv 1) \text{ independently } \text{ of } \mu \ \text{ if }\ \rho = \rho _s; \\&\text{ monotonically } \text{ decreasing } \text{ in } \text{ terms } \text{ of } \mu \ \text{ if }\ \rho < \rho _s. \end{aligned} \right.$$
- $G(\mu , \rho ) < 1$ for any positive $\mu$ and $\rho < \rho _s$ if and only if $\epsilon < 1/(1+c)$.
- $$G(\mu , 0)$$ is $$\left\{ \begin{aligned}&\text{ monotonically } \text{ increasing } \text{ in } \text{ terms } \text{ of } \mu \ \text{ if }\ \rho _s< 0,\ \text{ that } \text{ is },\ \epsilon> \dfrac{1}{1+c}; \\&\text{ constant } (\equiv 1) \text{ independently } \text{ of } \mu \ \text{ if }\ \rho _s = 0,\ \text{ that } \text{ is },\ \epsilon = \dfrac{1}{1+c}; \\&\text{ monotonically } \text{ decreasing } \text{ in } \text{ terms } \text{ of } \mu \ \text{ if }\ \rho _s > 0,\ \text{ that } \text{ is },\ \epsilon < \dfrac{1}{1+c}. \end{aligned} \right.$$
- $$G(\mu , 0) \left\{ \begin{aligned}&>1 \ \text{ for } \text{ any }\ \mu> 0 \ \text{ and }\ \epsilon> \dfrac{1}{1+c}; \\&>1 \ \text{ for } \text{ any }\ \mu > 0 \ \text{ and }\ \epsilon < \dfrac{1}{1+c}. \end{aligned} \right.$$
- $$\displaystyle \inf _{\mu \in (0,\infty )}G(\mu , \rho ) = \left\{ \begin{array}{l} G(0, \rho ) = 1\ \text{ for }\ \epsilon \ge \dfrac{1}{1+c}; \\ \displaystyle \lim _{\mu \rightarrow \infty }G(\mu , \rho ) = G_\infty (\rho ) := \dfrac{1+c}{(1-\rho )/\epsilon +\rho }< 1 \ \text{ for }\ \epsilon < \dfrac{1}{1+c}. \end{array} \right.$$
- $$\displaystyle \sup _{\mu \in (0,\infty )}G(\mu , \rho ) = \left\{ \begin{array}{l} G_\infty (\rho ) \ge 1\ \text{ for }\ \rho \ge \rho _s; \\ G(0, \rho ) = 1\ \text{ for }\ \rho < \rho _s. \end{array} \right.$$
- $$\displaystyle \inf _{(0, \infty )\times (0, 1)}G(\mu , \rho ) = \left\{ \begin{array}{l} G(0, 0) = 1\ \text{ for }\ \epsilon \ge \dfrac{1}{1+c}; \\ \displaystyle \lim _{\mu \rightarrow \infty }G(\mu , 0) = \epsilon (1+c)< 1\ \text{ for }\ \epsilon < \dfrac{1}{1+c}. \end{array} \right.$$
- $$\displaystyle \sup _{(0, \infty )\times (0, 1)}G(\mu , \rho ) = \lim _{\mu \rightarrow \infty }G(\mu , 1) = 1+c.$$

**Fig. 5:**
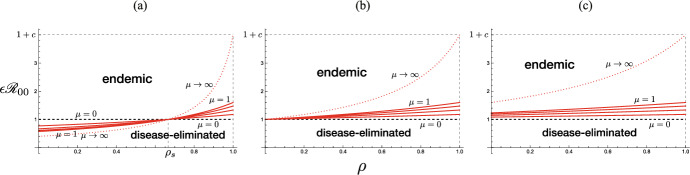
Parameter region and boundary indicated by the condition (9). The boundary curve is given by $G(\mu , \rho )$. **a** $\epsilon < 1/(1+c)$; **b** $\epsilon = 1/(1+c)$; **c** $\epsilon > 1/(1+c)$. Numerically drawn with **a** $\epsilon = 0.10$; **b** $\epsilon = 0.25$; **c** $\epsilon = 0.40$, commonly for $c = 3.0$. Solid curves are for $\mu = 0.25,\, 0.5,\, 0.75,\, 1.0$ in each figure. Dotted curve indicates $G_\infty (\rho )$

**Fig. 6:**
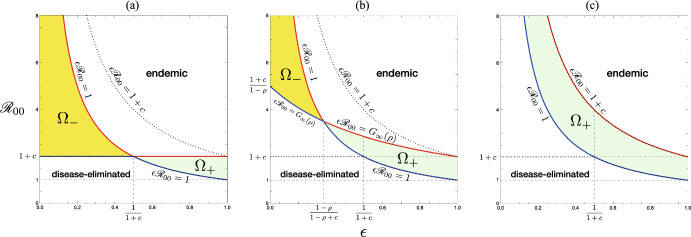
$(\epsilon , {\mathscr {R}}_{00})$-dependence of the endemicity, derived from the condition (12) in Theorem 9.1. Numerically drawn for **a** $\rho = 0.0$; **b** $\rho = 0.6$; **c** $\rho = 1.0$, commonly with $c = 1.0$. For the region $\Omega _+$, the acceptance of visitors may change the endemic situation of the community for the disease-eliminated equilibrium as described in Theorem 9.2, while, for the region $\Omega _-$, it may drive the situation of the community approaching the disease-eliminated equilibrium toward the endemic equilibrium as described in Theorem 9.3. For the region out of $\Omega _-$ and $\Omega _+$, the endemicity is independent of whether the community accepts visitors or not

Then from these mathematical features of $G(\rho , \mu )$, and Theorems 8.1, 8.2, and 8.3, we can get the following result on the disease endemicity in the community accepting the visitors (see Figs. 5 and 6):

#### Theorem 9.1

Independently of whether the community accepts the visitors or not, it approaches an endemic equilibrium if $\epsilon {\mathscr {R}}_{00} \ge \max \big [1,\, G_\infty (\rho )\big ],$ while it approaches the disease-eliminated equilibrium if $\epsilon {\mathscr {R}}_{00} \le \min \big [G_\infty (\rho ), 1\big ]$, where

$$\begin{aligned} G_\infty (\rho ):= \displaystyle \lim _{\mu \rightarrow \infty }G(\mu , \rho ) = \dfrac{1+c}{(1-\rho )/\epsilon +\rho }. \end{aligned}$$

Only when

12 $$\begin{aligned} \min \big [G_\infty (\rho ), 1\big ]< \epsilon {\mathscr {R}}_{00} < \max \big [1,\, G_\infty (\rho )\big ], \end{aligned}$$

the endemicity could significantly depend on the acceptance of visitors.

**Fig. 7:**
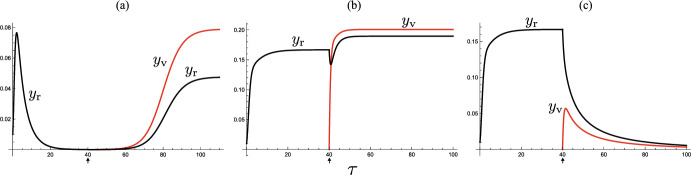
Temporal variations of infective subpopulations $y_{\textrm{r}}$ and $y_{\textrm{v}}$ by the systems (5) and (8). Numerically drawn for model (8) until $\tau = 40$ and model (5) for $\tau > 40$, with **a** $(\epsilon , \mu , \rho ) = (0.2, 0.9, 0.3)$ (${\mathscr {R}}_0 = 3.55$; $\epsilon {\mathscr {R}}_{00} = 0.8$); **b** $(\epsilon , \mu , \rho ) = (0.3, 0.9, 0.3)$ (${\mathscr {R}}_0 = 3.60$; $\epsilon {\mathscr {R}}_{00} = 1.2$); **c** $(\epsilon , \mu , \rho ) = (0.3, 0.9, 0.9)$ (${\mathscr {R}}_0 = 2.81$; $\epsilon {\mathscr {R}}_{00} = 1.2$); and commonly $\mathscr {R}_{00} = 4.0$; $c = 1.0$; $\omega = 1.0$; $(x_{\textrm{r}}(0), y_{\textrm{r}}(0) ) = (0.99, 0.01)$; $(x_{\textrm{v}}(40), y_{\textrm{v}}(40)) = (1-\rho , 0.0)$. In **a** and **c**, the endemicity is changed before and after starting the acceptance of visitors, while in **b** the system remains at an endemic state before and after it

The inequality (12) gives a necessary condition for which the acceptance of visitors could change the epidemic situation in the community from the endemic equilibrium to the disease-eliminated equilibrium or vice versa. The corresponding parameter regions are shown as $\Omega _-$ and $\Omega _+$ in Fig. 6. Numerical examples of such a change of endemicity by the acceptance of visitors are given in Fig. 7(a, c).

Further from the monotonicity of $G(\mu , \rho )$ in terms of $\mu$ and $\rho$ as described in the above, we find the following result on the condition with respect to the influence of the acceptance of visitors on the endemicity in the community:

#### Corollary 9.1.1

Independently of whether the community accepts the visitors or not, it approaches an endemic equilibrium if

$$\begin{aligned} \epsilon {\mathscr {R}}_{00} \ge \sup _{(0, \infty )\times (0, 1)}G(\mu , \rho ) = 1+c, \end{aligned}$$

while it approaches the disease-eliminated equilibrium if

$$\begin{aligned} \epsilon {\mathscr {R}}_{00} \le \inf _{(0, \infty )\times (0, 1)}G(\mu , \rho ) = \min \big [1,\ \epsilon (1+c)\big ]. \end{aligned}$$

Only when

13 $$\begin{aligned} \min \big [1,\ \epsilon (1+c)\big ]< \epsilon {\mathscr {R}}_{00} < 1+c, \end{aligned}$$

the endemicity could significantly depend on the acceptance of visitors.

As seen in Figs. 5 and 6, such an influence to cause a change of endemicity depends on the nature of accepted visitors (i.e., the number, the immune proportion, and the duration of stay).

Moreover, from the features of $G(\mu , \rho )$ described in the above, we find that, if $\epsilon {\mathscr {R}}_{00} \ge G_\infty (\rho )\ge 1$, the disease is endemic independently of how many visitors the community accepts even under the condition (13), as seen in Fig. 5. Thus, in comparison to the result for the community with no visitor (i.e., $\mu = 0$) given by Theorem 7.1, we can get the following result (see Fig. 6):

#### Theorem 9.2

Suppose that the disease was endemic under the condition that $1<\epsilon {\mathscr {R}}_{00} <1+c$ before the community accepts visitors. If the community accepts visitors with an immune proportion

14 $$\begin{aligned} \rho \le \rho _\infty :=\dfrac{1}{1-\epsilon }\Big (1-\dfrac{1+c}{\ {\mathscr {R}}_{00}}\Big ), \end{aligned}$$

the disease remains endemic independently of how many visitors are accepted. If the community accepts visitors with an immune proportion $\rho > \rho _\infty$, then the acceptance of visitors so many as

15 $$\begin{aligned} \mu > \mu _c := \dfrac{1-1/(\epsilon {\mathscr {R}}_{00})}{1/(\epsilon \mathscr {R}_{00})-\{(1-\rho )/\epsilon +\rho \}/(1+c)} \end{aligned}$$

makes the community approach the disease-eliminated equilibrium. Even if $\rho > \rho _\infty$, the acceptance of visitors with $\mu \le \mu _c$ does not sufficiently shift the endemicity, and the disease remains endemic.

**Fig. 8:**
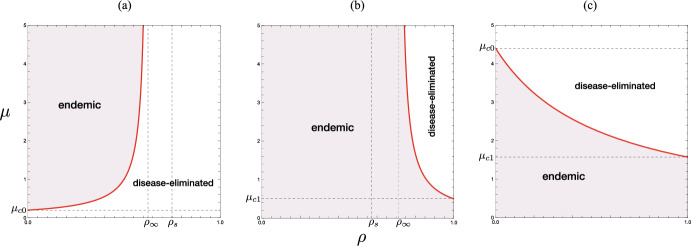
$(\rho , \mu )$-dependence of the endemicity, derived from the condition (9) with the results given by Theorems 8.1, 8.2, and 8.3: **a**, **b** $\epsilon < 1/(1+c)$; **c** $\epsilon > 1/(1+c)$. Numerically drawn for **a** $({\mathscr {R}}_{00}, \epsilon , c) = (4.0, 0.2, 1.0)$ ($\epsilon {\mathscr {R}}_{00} = 0.8$); **b** $({\mathscr {R}}_{00}, \epsilon , c) = (4.0, 0.3, 1.0)$ ($\epsilon {\mathscr {R}}_{00} = 1.2$); **c** $({\mathscr {R}}_{00}, \epsilon , c) = (1.8, 0.8, 1.0)$ ($\epsilon {\mathscr {R}}_{00} = 1.44$), each of which satisfies the condition (13) in Corollary 9.1.1

The critical value $\rho _\infty$ satisfies the equation $\epsilon {\mathscr {R}}_{00} = G_\infty (\rho _\infty )$. The latter case of $\rho > \rho _\infty$ in Theorem 9.2 corresponds to the parameter region $\Omega _+$ in Fig. 6. Figure 8(b) shows a numerical example of the $(\rho , \mu )$-dependence in such a case when $\epsilon {\mathscr {R}}_{00} >1$.

Since $\mu _c$ defined by (15) is monotonically decreasing in terms of $\rho$ when $\epsilon {\mathscr {R}}_{00} > 1$, we note that

16 $$\begin{aligned} \mu _c \ge \mu _c\big \vert _{\rho = 1} = \mu _{c1} := \dfrac{1-1/(\epsilon {\mathscr {R}}_{00})}{1/(\epsilon \mathscr {R}_{00})-1/(1+c)}, \end{aligned}$$

where $\mu _{c1} > 0$ for $1<\epsilon {\mathscr {R}}_{00} <1+c$. Then we get the following corollary:

#### Corollary 9.2.1

When $1<\epsilon {\mathscr {R}}_{00} <1+c$, if the community accepts visitors few enough to have $\mu < \mu _{c1}$, the disease remains endemic independently of how much proportion of visitors is immune at the entry.

This result is numerically pointed out in Fig. 8(b, c).

Moreover we note that, when ${\mathscr {R}}_{00}< 1+c$ so that $\rho _\infty$ defined by (14) is negative, the first case in Theorem 9.2 does not occur. Then the $(\rho , \mu )$-dependence becomes as shown by Fig. 8(c), where there is a finite value of $\mu$ beyond which the community approaches the disease-eliminated equilibrium, independently of the immune proportion in the visitors at the entry:

17 $$\begin{aligned} \mu _{c0} := \mu _c\big \vert _{\rho = 0} = \dfrac{1-1/(\epsilon \mathscr {R}_{00})}{1/(\epsilon {\mathscr {R}}_{00})-1/\{\epsilon (1+c)\}}. \end{aligned}$$

#### Corollary 9.2.2

When $1< \epsilon {\mathscr {R}}_{00} < \epsilon (1+c)$, if the community accepts visitors so many as $\mu > \mu _{c0}$, the disease tends to get eliminated independently of how much proportion of visitors is immune at the entry. When $\epsilon (1+c)\le \epsilon {\mathscr {R}}_{00} <1+c$, only the acceptance of visitors with $\rho >\rho _\infty$ and $\mu >\mu _c$ can change the endemicity and lead the community to the disease-eliminated equilibrium.

Hence the value $\mu _{c0}$ gives a sufficient number of accepted visitors which is effective to suppress the disease spread in the community when $1< \epsilon {\mathscr {R}}_{00} < \epsilon (1+c)$. See the numerical examples in Fig. 7(b, c).

In contrast, when the risk of reinfection is so weak as $\epsilon < 1/(1+c)$, the acceptance of visitors may cause the opposite influence on the epidemic dynamics, as numerically indicated by Fig. 8(a):

#### Theorem 9.3

Suppose that the disease was getting eliminated under the condition that $\epsilon (1+c)<\epsilon {\mathscr {R}}_{00} <1$ before the community accepts visitors. If the community accepts visitors with an immune proportion $\rho \ge \rho _\infty$, the disease keeps getting eliminated independently of how many visitors are accepted. If the community accepts visitors with an immune proportion $\rho < \rho _\infty$, then the acceptance of visitors so many as $\mu >\mu _c$ induces the endemicity, and the disease becomes endemic. Even if $\rho < \rho _\infty$, the acceptance of visitors so few as $\mu \le \mu _c$ does not induce the endemicity, and the disease keeps getting eliminated.

The situation considered in this theorem corresponds to the parameter region $\Omega _-$ in Fig. 6, and is numerically exemplified by Fig. 7(a). Theorem 9.3 indicates that, if the proportion of immune visitors is so low as $\rho < \rho _\infty$, there exists the upper threshold $\mu _c$ for the number of accepted visitors to suppress the revival of the disease spread after starting the acceptance of visitors in the community where the disease was getting eliminated.

**Fig. 9:**
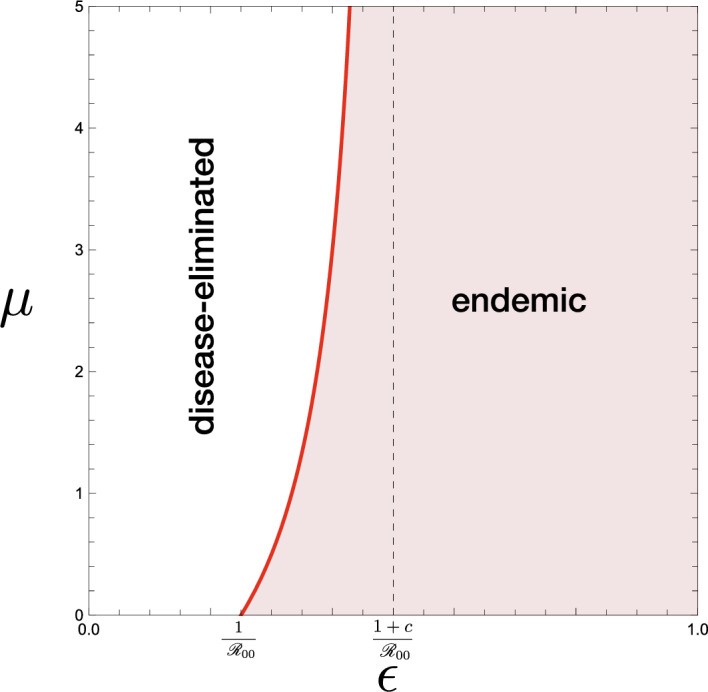
Parameter region and boundary indicated by the condition (9) with the results given by Theorems 8.1, 8.2, and 8.3 when the community accepts only immune visitors with $\rho = 1$. Numerically drawn with ${\mathscr {R}}_{00} = 4.0$ and $c = 1.0$. Refer to Sect. 9.2

### Acceptance of Only Immune Visitors

When the community accepts only immune visitors, that is, when $\mu > 0$ with $\rho = 1$, $G(\mu , 1)$ is necessarily greater than 1 and monotonically increasing in terms of $\mu$. Then, from Theorem 9.2, we can find that only the acceptance of visitors so many as $\mu > \mu _{c1}$ can induce the disease-eliminated equilibrium in the community where the disease was endemic before starting the acceptance of visitors. As defined by (16), the critical value $\mu _{c1}$ depends on the risk of reinfection, and then, from Theorems 9.1 and 9.2, we can obtain the following result (see Figs. 8 and 9):

#### Corollary 9.2.3

Suppose that the disease was endemic under the condition that $\epsilon {\mathscr {R}}_{00} >1$ before the community accepts only immune visitors. If $\epsilon {\mathscr {R}}_{00} \ge 1+c$, the endemicity remains independently of how many visitors the community accepts. If $1<\epsilon {\mathscr {R}}_{00} <1+c$, the acceptance of visitors so many as $\mu > \mu _{c1}$ is effective to make the disease eliminated.

Therefore the community under an endemic situation could have a preferable influence to suppress the endemicity by accepting only immune visitors only when the reinfectivity is sufficiently low as indicated by Fig. 9.

If the community was approaching the disease-eliminated equilibrium with the risk of reinfection so low as $\epsilon {\mathscr {R}}_{00} \le 1$, the community keeps approaching the disease-eliminated equilibrium even after starting the acceptance of only immune visitors, independently of how many visitors the community accepts.

### Acceptance of Only Susceptible Visitors

**Fig. 10:**
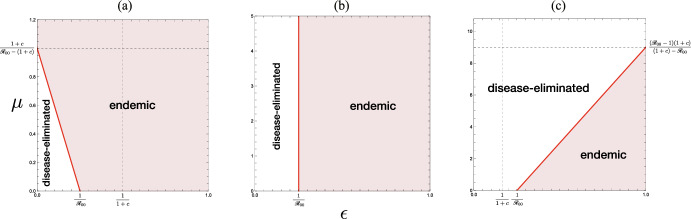
Parameter region and boundary indicated by the condition the condition (9) with the results given by Theorems 8.1, 8.2, and 8.3 when all visitors accepted by the community is susceptible with $\rho = 0$: **a** ${\mathscr {R}}_{00} > 1+c$; **b** ${\mathscr {R}}_{00} = 1+c$; **c** ${\mathscr {R}}_{00} < 1+c$. Numerically drawn with **a** $c=1.0$; **b** $c=3.0$; **c** $c=5.0$, and commonly ${\mathscr {R}}_{00} = 4.0$. Refer to Sect. 9.3

Now let us consider the case where all visitors accepted by the community are susceptible, that is, when $\mu > 0$ with $\rho = 0$. Then, from Theorem 9.3, we find three different cases according to the influence of the acceptance of visitors as shown in Fig. 10, taking account of the features of $G(\mu , 0)$ given in Sect. 9.1. We can get the following result:

#### Corollary 9.3.1

When the disease was getting eliminated under the condition that $\epsilon {\mathscr {R}}_{00} \le 1$, the acceptance of only susceptible visitors induces

$$\begin{aligned} \left\{ \begin{aligned}&\text{ no } \text{ endemicity } \text{ if }\ \epsilon {\mathscr {R}}_{00}\le \epsilon (1+c); \\&\text{ no } \text{ endemicity } \text{ if }\ \epsilon {\mathscr {R}}_{00}>\epsilon (1+c)\, \text{ and }\ \mu \le \mu _{c0}; \\&\text{ the } \text{ endemicity } \text{ if }\ \epsilon {\mathscr {R}}_{00}>\epsilon (1+c)\, \text{ and }\ \mu >\mu _{c0}. \end{aligned} \right. \end{aligned}$$

In contrast, when the disease was endemic under the condition that $\epsilon {\mathscr {R}}_{00} > 1$, the acceptance of only susceptible visitors induces

$$\begin{aligned} \left\{ \begin{aligned}&\text{ no } \text{ change } \text{ in } \text{ the } \text{ endemicity } \text{ if }\ \epsilon {\mathscr {R}}_{00}\ge \epsilon (1+c); \\&\text{ no } \text{ change } \text{ in } \text{ the } \text{ endemicity } \text{ if }\ \epsilon {\mathscr {R}}_{00}<\epsilon (1+c)\, \text{ and }\ \mu \le \mu _{c0}; \\&\text{ the } \text{ elimination } \text{ of } \text{ disease } \text{ if }\ \epsilon {\mathscr {R}}_{00}< \epsilon (1+c)\, \text{ and }\ \mu > \mu _{c0}. \end{aligned} \right. \end{aligned}$$

The critical value $\mu _{c0}$ is defined by (17).

Therefore the acceptance of only susceptible visitors could have the counter effect according to the endemicity, depending on the infectivity of disease. Only for a moderately high infectious disease such that $1/\epsilon<{\mathscr {R}}_{00}< 1+c$, the acceptance of only susceptible visitors so many as $\mu >\mu _{c0}$ can lead the community to the disease-eliminated equilibrium. For the disease with a low reinfectivity such that $1+c <\mathscr {R}_{00} \le 1/\epsilon$, the acceptance of only susceptible visitors so many as $\mu >\mu _{c0}$ can lead the community to the endemic equilibrium.

Further, as indicated by Fig. 10, we find that there is a sufficient value of $\mu$ which determines the epidemic situation in the community:

#### Corollary 9.3.2

If ${\mathscr {R}}_{00} > 1+c$, the acceptance of only susceptible visitors so many as

$$\begin{aligned} \mu \ge \dfrac{1+c}{{\mathscr {R}}_{00}-(1+c)} \end{aligned}$$

necessarily makes the disease endemic. In contrast, if $\mathscr {R}_{00} < 1+c$, the acceptance of only susceptible visitors so many as

$$\begin{aligned} \mu \ge \dfrac{({\mathscr {R}}_{00}-1)(1+c)}{(1+c)-{\mathscr {R}}_{00}} \end{aligned}$$

necessarily makes the disease eliminated.

The former case means an unpreferable influence of the sufficiently large number of visitors for the community with the spread of a highly infectious disease, while the latter does a preferable influence for the community with the spread of a moderately infectious disease.

**Fig. 11:**
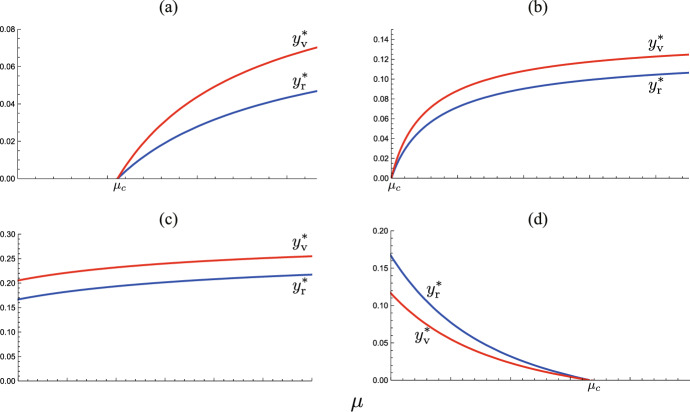
$\mu$-dependence of endemic sizes. Numerically drawn by (10) with **a** $(\epsilon , \rho ) = (0.2, 0.4)$ ($\epsilon {\mathscr {R}}_{00} = 0.8$, $\mu _c = 0.56$); **b** $(\epsilon , \rho ) = (0.25, 0.4)$ ($\epsilon {\mathscr {R}}_{00} = 1.0$, $\mu _c = 0.0$, $\rho _c = 0.67$), **c** $(\epsilon , \rho ) = (0.3, 0.1)$ ($\epsilon {\mathscr {R}}_{00} = 1.2$, $\mu _c <0$, $\rho _c = 0.40$), **d** $(\epsilon , \rho ) = (0.3, 0.8)$ ($\epsilon {\mathscr {R}}_{00} = 1.2$, $\mu _c = 1.67$, $\rho _c = 0.40$), and commonly ${\mathscr {R}}_{00} = 4.0$; $c = 1.0$

### Change in Endemic Size

Figure 11 shows the numerically drawn $\mu$-dependence of endemic sizes $y_{\textrm{r}}^*$ and $y_{\textrm{v}}^*$ at the endemic equilibrium determined by (10). As the figure implies, the endemic size necessarily has a monotonic dependence on the number of accepted visitors, represented now by $\mu$, about which we can get the following analytical result (Appendix G):

#### Theorem 9.4

The endemic sizes $y_{\textrm{r}}^*$, $y_{\textrm{v}}^*$, and the total endemic size

18 $$\begin{aligned} z^* := \dfrac{y_{\textrm{r}}^*+\mu y_{\textrm{v}}^*}{1+\mu } = \dfrac{I_{\textrm{r}}^*+I_{\textrm{v}}^*}{N+m} \end{aligned}$$

are monotonically increasing in terms of $\mu$ if and only if $\epsilon {\mathscr {R}}_{00}\le 1$ or

19 $$\begin{aligned} \left\{ \begin{aligned} \ {}&\epsilon {\mathscr {R}}_{00} > 1; \\ \ {}&\rho <\rho _c := \dfrac{1-\epsilon ^2{\mathscr {R}}_{00}-\epsilon c}{(1-\epsilon )\epsilon {\mathscr {R}}_{00}}. \end{aligned} \right. \end{aligned}$$

**Fig. 12:**
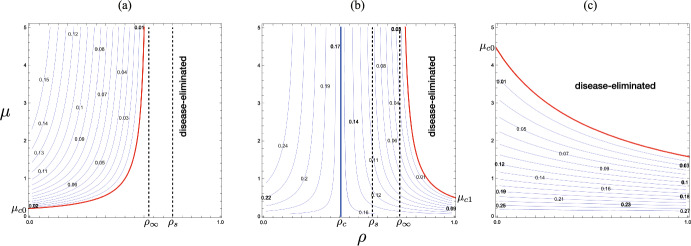
$(\rho , \mu )$-dependence of the endemic size $y_{\textrm{r}}^*$. Numerically drawn contour maps for three cases correspond to those in Fig. 8: **a** $\epsilon {\mathscr {R}}_{00} = 0.8$; **b** $\epsilon {\mathscr {R}}_{00} = 1.2$ and $\rho _c = 0.40$; **c** $\epsilon {\mathscr {R}}_{00} = 1.44$ and $\rho _c = -3.31$, where the parameter values are respectively the same as in Fig. 8

It can be easily found that $\rho _c < 1$ when $\epsilon {\mathscr {R}}_{00} > 1$. See the numerically drawn $(\rho , \mu )$-dependence of the endemic size $y_{\textrm{r}}^*$ in Fig. 12.

We remark that, as shown in Sect. 9.2, the endemic equilibrium for $\rho =1$ exists only when $\epsilon {\mathscr {R}}_{00} > 1$. Then we can find the following result too (Appendix H):

#### Corollary 9.4.1

When the community accepts only immune visitors (i.e., $\rho = 1$), the endemic size is monotonically decreasing in terms of $\mu$.

This result may be regarded as included in Theorem 9.4 because the condition given in Theorem 9.4 can never hold when $\rho =1$. We can see the numerical examples in Fig. 12(b, c).

When $\rho _c$ defined by (19) is non-positive with $\epsilon {\mathscr {R}}_{00} > 1$, any $\rho$ cannot be smaller than $\rho _c$, so that the endemic size is necessarily monotonically decreasing in terms of $\mu$:

#### Corollary 9.4.2

If $\epsilon {\mathscr {R}}_{00} > \max \big [\, 1, 1/\epsilon -c\,\big ]$, the endemic size is necessarily monotonically decreasing in terms of $\mu$.

The numerical example of Fig. 12(c) illustrates the case.

For the critical case of $\rho = \rho _c > 0$ with $\epsilon \mathscr {R}_{00} > 1$, we can derive the explicit values at the endemic equilibrium $E_{00}$ from (10) (Appendix G):

20 $$\begin{aligned} x_{\textrm{v}}^*=\dfrac{c}{(1-\epsilon ){\mathscr {R}}_{00}}; \ y_{\textrm{v}}^*=y_{\textrm{r}}^* = z^* = 1-\dfrac{1}{\epsilon {\mathscr {R}}_{00}}. \end{aligned}$$

Hence the endemic sizes are independent of the number of accepted visitors in this case:

#### Corollary 9.4.3

For $\rho = \rho _c > 0$ with $\epsilon {\mathscr {R}}_{00} > 1$, the endemic sizes $y_{\textrm{r}}^*$, $y_{\textrm{v}}^*$, and the total endemic size $z^*$ are determined independently of $\mu$.

A numerical example is given in Fig. 12(b). We note it necessary for $\rho _c > 0$ with $\epsilon {\mathscr {R}}_{00} > 1$ that $1/\epsilon - c > 1$, that is, $\epsilon (1+c) < 1$. Moreover, the case of Corollary 9.4.3 can appear only when $1<\epsilon {\mathscr {R}}_{00} < 1/\epsilon - c$.

Additionally we can find the following relations among the specific values $\rho _s$, $\rho _\infty$, and $\rho _c$ for the immune proportion of accepted visitors at the entry:

#### Corollary 9.4.4

It holds that $\rho _c < \rho _\infty$ and $\rho _c < \rho _s$.

The proof is easy by calculating the differences $\rho _\infty - \rho _c$ and $\rho _s -\rho _c$ to show them positive. Numerical calculation of Fig. 12(b) demonstrates this result.

**Fig. 13:**
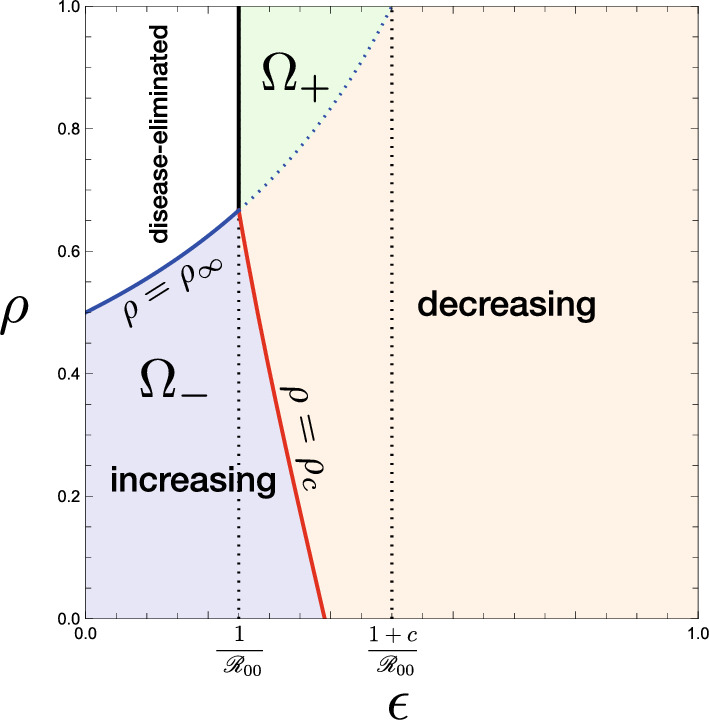
Classification of the parameter region of $(\epsilon , \rho )$ according to the $\mu$-dependence of the change in the endemic size. Numerically drawn with ${\mathscr {R}}_{00} = 4.0$ and $c = 1.0$. Regions $\Omega _\pm$ correspond to those in Fig. 6

Consequently as indicated by Fig. 13, the larger number of accepted visitors makes the endemic size bigger only when the immune proportion of accepted visitors at the entry is sufficiently small under the epidemic situation with a sufficiently low risk of infection.

## Concluding Remarks

The results of our model imply that the acceptance of temporal visitors from the outside may induce a significant change of the epidemic state in the community. Contrary to an intuitive expectation, the acceptance of visitors does not necessarily make the epidemic situation worse in the community. Only when the reinfectivity of the disease is sufficiently weak, the acceptance of visitors may induce the endemicity if the community accepts the visitors with a sufficiently low immune proportion. Furthermore, when the reinfectivity is high, the acceptance of a sufficiently large number of visitors may induce the elimination of the disease if the community can regulate to accept the visitors with a sufficiently high immune proportion.

The visitors certainly play a role of recruitment of hosts for the infectious disease spread in the community. The visitors with a higher susceptible proportion could be regarded as a larger supply of susceptible individuals in the community, and they subsequently provide a fast recruitment of infectives. In contrast, the visitors with a high immune proportion cause only a slow recruitment of infectives with the reinfection. For these reasons, the influence of the visitor acceptance on the epidemic dynamics with a reinfectious disease must depend on the immune proportion in the visitors at the entry. On the other hand, the entry of many visitors could induce a dilution of the infective density in the community at the same time, which is regarded as an advantageous influence of the visitor acceptance against the disease spread. In the epidemic dynamics with our model, a balance of these counteractive factors of the visitor acceptance with respect to the disease spread could significantly affect the consequence of epidemic dynamics in the community.

As a result, a preferable acceptance of visitors must be regulated to have a sufficiently large immune proportion according to the public health in the community. In this sense, the best policy for the visitor acceptance would be to allow the entry only for the immune visitors. From the results on our model, such an acceptance of only immune visitors may lower the endemic size, and further suppress the endemicity to induce the elimination of the disease spread in the community.

In contrast, when the community was on the way to the disease-eliminated equilibrium before starting the acceptance of visitors, the acceptance of visitors without any epidemiological regulation may cause the revival of the disease spread in the community. Such a case would occur by reduced cautiousness of the disease before starting the acceptance of visitors, which could be caused by the reason that the number of infective residents became rather small in comparison to that at the outbreak.

Our model could be regarded as a consideration on the epidemic dynamics in a season. In this sense, the number of visitors may be beyond the number of residents in the community (i.e., $\mu > 1$), as some popular touristic local places like Venice in the vacation season, or a certain place attracting visitors like a newly found gold mine. As another example, we could consider a community accepting many evacuees from a certain calamity. Even though the number of visitors would be smaller than the number of residents in most cases (i.e., $\mu < 1$), our results imply that the influence of the visitor acceptance could depend on the infectivity and reinfectivity in the epidemic dynamics, and the regulation on the epidemiological nature of accepted visitors.

Since the infectivity and reinfectivity are not only determined by the nature of disease itself but also by social custom, sanitary condition, and people’s behavior (Ferguson et al. 2005; Heymann 2005; Funk et al. 2009; Perisic and Bauch 2009; Manfredi and D’Onofrio 2013; Bavel et al. 2020; Kapitány-Fövény and Sulyok 2020; Thu et al. 2020), the influence of the visitor acceptance could depend also on social factors in the community which accepts the visitors under the epidemic dynamics. Such social factors could be affected by the situation of disease spread during the epidemic dynamics in the community. For example, some strategic/non-strategic transmission of information about the disease spread or a public health campaign to prevent the further disease spread could alter people’s social behavior, and subsequently the risk of infection/reinfection. Hence if the infectivity and reinfectivity would be changeable in the epidemic dynamics, the influence of the visitor acceptance must be qualitatively changed.

The results from our model imply such a possibility that a shift of the infectivity and/or reinfectivity to the weaker would induce an epidemic situation in which the acceptance of visitors causes the increase in the epidemic size or the revival of disease spread even with the endemicity. If so, there would be repetitive revivals of disease spread in the community, driven by a temporal shift of the infectivity and/or reinfectivity which could bring a feedback influence on the policy to control the disease spread in the community. Such theoretical/mathematical researches on the relation between the disease spread and the nature of hosts are interesting and require further development.

## Figures and Tables

**Fig. 14:**
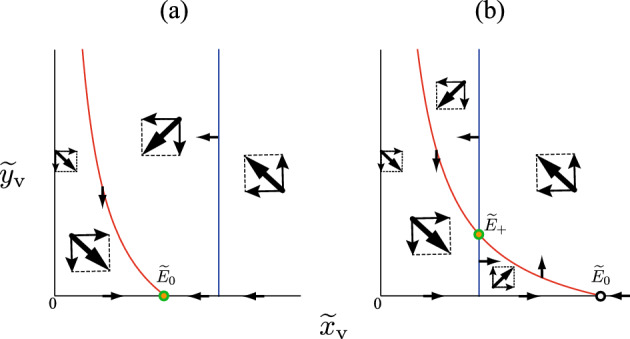
Application of the isocline method for the system (B1) when the condition (7) is (a) not satisfied; (b) satisfied

## Data Availability

Not applicable.

## Appendix A Derivation of ${\mathscr {R}}_0$

In our model, the new cases consist of residents and visitors. Therefore, from the conceptual definition and the mathematical feature of the basic reproduction number, we shall derive it here from the following conditions:

$$\begin{aligned} \left. \dfrac{d(I_{\textrm{v}}+I_{\textrm{r}})}{dt} \right| _{0< I_{\textrm{v}}+I_{\textrm{r}}\ll 1}> 0 \ \text{ for }\ {\mathscr {R}}_0 > 1; \ \left. \dfrac{d(I_{\textrm{v}}+I_{\textrm{r}})}{dt} \right| _{0< I_{\textrm{v}}+I_{\textrm{r}}\ll 1}< 0 \ \text{ for }\ {\mathscr {R}}_0 < 1, \end{aligned}$$

for the initial condition (3) with $0<I_{\textrm{v}}(0)+I_{\textrm{r}}(0) =0+ I_{\textrm{r}0}\ll 1$. This is because the basic reproduction number is defined as the expected number of new infectives produced by one infective individual in an environment consisting of only susceptibles. Following the assumption H3 in Sect. 2, we shall adopt the initial condition (3) in order to define the basic reproduction number. Then, in place of the above conditions, we can use the followings:

$$\begin{aligned} \left. \dfrac{d(I_{\textrm{v}}+I_{\textrm{r}})}{dt} \right| _{t = 0}> 0 \ \text{ for }\ {\mathscr {R}}_0 > 1; \ \left. \dfrac{d(I_{\textrm{v}}+I_{\textrm{r}})}{dt} \right| _{t = 0}< 0 \ \text{ for }\ {\mathscr {R}}_0 < 1 \end{aligned}$$

for the initial condition (3) with $0<I_{\textrm{v}}(0)+I_{\textrm{r}}(0) =0+ I_{\textrm{r}0}\ll 1$. Remark that, in this context about the situation to define the basic reproduction number of our model, the initial infective must be a resident, which matches the assumption H3 in Sect. 2. Then, making use of (3), we have

$$\begin{aligned} \left. \dfrac{d(I_{\textrm{v}}+I_{\textrm{r}})}{dt} \right| _{t = 0}&= \Big \{ \beta \dfrac{(1-\rho )m+S_{\textrm{r}0}}{N+m} +\epsilon \beta \dfrac{\rho m+R_{\textrm{r}0}}{N+m} -\gamma \Big \} I_{\textrm{r}0}. \end{aligned}$$

Thus we find that

$$\begin{aligned} \left. \dfrac{d(I_{\textrm{v}}+I_{\textrm{r}})}{dt} \right| _{t=0}> 0&\ \text{ if } \text{ and } \text{ only } \text{ if }\ \dfrac{\, \beta \, }{\gamma }\dfrac{(1-\rho )m+S_{\textrm{r}0}}{N+m} +\dfrac{\epsilon \beta }{\gamma }\dfrac{\rho m+R_{\textrm{r}0}}{N+m} > 1. \end{aligned}$$

Consequently we can define the basic reproduction number $\mathscr {R}_0$ as follows:

$$\begin{aligned} {\mathscr {R}}_0&= \sup _{(S_{\textrm{r}0},R_{\textrm{r}0})} \Big \{ \dfrac{\, \beta \, }{\gamma }\dfrac{(1-\rho )m+S_{\textrm{r}0}}{N+m} +\dfrac{\epsilon \beta }{\gamma }\dfrac{\rho m+R_{\textrm{r}0}}{N+m} \Big \} \\&= \sup _{S_{\textrm{r}0}} \Big \{ \dfrac{\, \beta \, }{\gamma }\dfrac{(1-\rho )m+S_{\textrm{r}0}}{N+m} +\dfrac{\epsilon \beta }{\gamma }\dfrac{\rho m+N-S_{\textrm{r}0}}{N+m} \Big \} \\&= \sup _{S_{\textrm{r}0}} \Big \{ \dfrac{\, \beta \, }{\gamma }\dfrac{(1-\rho )m}{N+m} +\dfrac{\epsilon \beta }{\gamma }\dfrac{\rho m+N}{N+m} +\dfrac{(1-\epsilon )\beta }{\gamma }\dfrac{S_{\textrm{r}0}}{N+m} \Big \} \\&= \dfrac{\, \beta \, }{\gamma }\dfrac{(1-\rho )m+N}{N+m} +\dfrac{\epsilon \beta }{\gamma }\dfrac{\rho m}{N+m}. \end{aligned}$$

This formula can be rewritten as given by (4) to clarify the meaning.

## Appendix B Proof for Theorem 6.1

From the arguments in the first paragraph of Sect. 6, we find that the dynamics given by (5) with $\epsilon = 0$ necessarily approaches the dynamics with the following limiting system in terms of the visitor population:

B1 $$\begin{aligned} \begin{aligned} \dfrac{d{{\widetilde{x}}}_{\textrm{v}}}{d\tau }&= (1-\rho )c -\mathscr {R}_{00}\dfrac{\mu }{1+\mu }{{\widetilde{y}}}_{\textrm{v}}\widetilde{x}_{\textrm{v}} -c{{\widetilde{x}}}_{\textrm{v}} ; \\ \dfrac{d{{\widetilde{y}}}_{\textrm{v}}}{d\tau }&= \mathscr {R}_{00}\dfrac{\mu }{1+\mu }{{\widetilde{y}}}_{\textrm{v}}\widetilde{x}_{\textrm{v}} - (1+c ){{\widetilde{y}}}_{\textrm{v}}. \end{aligned} \end{aligned}$$

The feasible equilibria are ${{\widetilde{E}}}_0(1-\rho ,\, 0)$ and ${{\widetilde{E}}}_+({{\widetilde{x}}}_{\textrm{v}}^*,\, {{\widetilde{y}}}_{\textrm{v}}^*)$ with (6). The former ${{\widetilde{E}}}_0$ corresponds to the disease-eliminated equilibrium for the system (5), $E_{00}(1-\rho , 0, 0, 0)$, and so does the latter ${{\widetilde{E}}}_+$ to the endemic equilibrium $E_{+0}({{\widetilde{x}}}_{\textrm{v}}^*,\, {{\widetilde{y}}}_{\textrm{v}}^*, 0, 0)$. The endemic equilibrium $E_{+0}$ can exist when and only when the condition (7) is satisfied. By the local stability analysis with the eigenvalues of the Jacobi matrix at the equilibrium, we can easily find that the endemic equilibrium $E_{+0}$ is locally asymptotically stable when it exists. In the following part, we shall consider its global stability.

First we set the following mathematical result on the boundedness for the solution of the system (B1):

### Lemma B.1

For any initial condition $\big ({{\widetilde{x}}}_{\textrm{v}}(0), {{\widetilde{y}}}_{\textrm{v}}(0)\big )$ in the domain

B2 $$\begin{aligned} D:=\Big \{ \big ({{\widetilde{x}}}_{\textrm{v}}, {{\widetilde{y}}}_{\textrm{v}}\big ) \mid {{\widetilde{x}}}_{\textrm{v}}>0,\ {{\widetilde{y}}}_{\textrm{v}} > 0,\ {{\widetilde{x}}}_{\textrm{v}}+{{\widetilde{y}}}_{\textrm{v}} < 1-\rho \, \Big \}, \end{aligned}$$

the solution $\big ({{\widetilde{x}}}_{\textrm{v}}(\tau ), \widetilde{y}_{\textrm{v}}(\tau )\big )$ of (B1) stays in *D* for any $\tau > 0$.

### Proof

We can obtain the following features from (B1) for the initial condition $\big (\widetilde{x}_{\textrm{v}}(0), {{\widetilde{y}}}_{\textrm{v}}(0)\big )\in D$:

$$\begin{aligned}&\left. \dfrac{d{{\widetilde{x}}}_{\textrm{v}}}{d\tau }\right| _{x_{\textrm{v}} = 0} = 1-\rho> 0; \ \widetilde{y}_{\textrm{v}}(\tau ) = {{\widetilde{y}}}_{\textrm{v}}(0) \exp \left[ \displaystyle \int _0^\tau \Big \{ \mathscr {R}_{00}\dfrac{\mu }{1+\mu }{{\widetilde{x}}}_{\textrm{v}}(s)- (1+c ) \Big \} ds \right] >0. \end{aligned}$$

The first inequality indicates that ${{\widetilde{x}}}_{\textrm{v}}$ cannot reach 0 from the initial value ${{\widetilde{x}}}_{\textrm{v}}(0) > 0$. The second equation indicates that ${{\widetilde{y}}}_{\textrm{v}}$ is necessarily positive for any $\tau > 0$ and ${{\widetilde{y}}}_{\textrm{v}}(0) > 0$. Then, from

$$\begin{aligned} \left. \dfrac{d\big ({{\widetilde{x}}}_{\textrm{v}}+{{\widetilde{y}}}_{\textrm{v}}\big )}{d\tau } \right| _{{{\widetilde{x}}}_{\textrm{v}} +\widetilde{y}_{\textrm{v}}= 1-\rho } = -{{\widetilde{y}}}_{\textrm{v}} < 0 \end{aligned}$$

for ${{\widetilde{y}}}_{\textrm{v}} > 0$, we can find that $\widetilde{x}_{\textrm{v}}+{{\widetilde{y}}}_{\textrm{v}}$ cannot become $\widetilde{x}_{\textrm{v}}+{{\widetilde{y}}}_{\textrm{v}} = 1-\rho$ for any $\tau > 0$ and initial condition in *D*. $\square$

Lemma B.1 means that the domain *D* is invariant for the dynamics given by (B1). Further, when the endemic equilibrium ${{\widetilde{E}}}_+$ exists, satisfying the condition (7), we find from (6) that

$$\begin{aligned} {{\widetilde{x}}}_{\textrm{v}}^* +{{\widetilde{y}}}_{\textrm{v}}^*&= (1-\rho )\dfrac{c}{c+1}+\dfrac{1+\mu }{\mu }\dfrac{1}{{\mathscr {R}}_{00}} \\&< (1-\rho )\dfrac{c}{c+1}+\dfrac{1+\mu }{\mu }\dfrac{1}{c+1}(1-\rho )\dfrac{\mu }{1+\mu } = 1-\rho . \end{aligned}$$

Hence we have the following result:

### Lemma B.2

When ${{\widetilde{E}}}_+({{\widetilde{x}}}_{\textrm{v}}^*,\, \widetilde{y}_{\textrm{v}}^*)$ exists, it must belong to the domain *D* defined by (B2).

When the condition (7) is not satisfied, that is, when the endemic equilibrium does not exist, we can easily find that the disease-eliminated equilibrium is globally asymptotically stable, making use of the isocline method shown by Fig. 14(a). In contrast, as seen from Fig. 14(b), when the condition (7) is satisfied and the endemic equilibrium ${{\widetilde{E}}}_+$ exists, the stability cannot be determined only by the isocline method.

When the endemic equilibrium ${{\widetilde{E}}}_+$ exists, let us consider the following function of $({{\widetilde{x}}}_{\textrm{v}},\, {{\widetilde{y}}}_{\textrm{v}})$ in the domain *D* defined by Lemma B.1:

B3 $$\begin{aligned} V\big ({{\widetilde{x}}}_{\textrm{v}},\, {{\widetilde{y}}}_{\textrm{v}}\big )&:= \dfrac{\, 1\, }{2} \big \{ ({{\widetilde{x}}}_{\textrm{v}}^* - {{\widetilde{x}}}_{\textrm{v}}) + ({{\widetilde{y}}}_{\textrm{v}}^* - \widetilde{y}_{\textrm{v}}) \big \}^2 \nonumber \\&\quad + \Big ( {{\widetilde{x}}}_{\textrm{v}}^* + c\dfrac{1+\mu }{{\mathscr {R}}_{00}\mu } \Big ) \Big ( \widetilde{y}_{\textrm{v}} -{{\widetilde{y}}}_{\textrm{v}}^* - {{\widetilde{y}}}_{\textrm{v}}^* \ln \dfrac{\ {{\widetilde{y}}}_{\textrm{v}}}{\ {{\widetilde{y}}}_{\textrm{v}}^*} \Big ). \end{aligned}$$

It can be easily found that $V\big ({{\widetilde{x}}}_{\textrm{v}}^*,\, {{\widetilde{y}}}_{\textrm{v}}^*\big ) = 0$ and $V\big (\widetilde{x}_{\textrm{v}},\, {{\widetilde{y}}}_{\textrm{v}}\big ) > 0$ for any $\big ({{\widetilde{x}}}_{\textrm{v}},\, {{\widetilde{y}}}_{\textrm{v}}\big ) \ne \big ({{\widetilde{x}}}_{\textrm{v}}^*,\, {{\widetilde{y}}}_{\textrm{v}}^*\big )$ in *D*. Further, making use of (B1), we can derive

$$\begin{aligned} \dfrac{dV}{d\tau } = - \big ( {{\widetilde{x}}}_{\textrm{v}}^*-\widetilde{x}_{\textrm{v}}\big )^2 - \dfrac{\mu }{1+\mu } {{\widetilde{x}}}_{\textrm{v}}^* \big ( {{\widetilde{y}}}_{\textrm{v}}^*-{{\widetilde{y}}}_{\textrm{v}}\big )^2, \end{aligned}$$

which becomes zero only for $\big ({{\widetilde{x}}}_{\textrm{v}},\, {{\widetilde{y}}}_{\textrm{v}}\big ) = \big ({{\widetilde{x}}}_{\textrm{v}}^*,\, {{\widetilde{y}}}_{\textrm{v}}^*\big )$, and negative for any $\big ({{\widetilde{x}}}_{\textrm{v}},\, {{\widetilde{y}}}_{\textrm{v}}\big ) \ne \big ({{\widetilde{x}}}_{\textrm{v}}^*,\, {{\widetilde{y}}}_{\textrm{v}}^*\big )$ in *D*. These features of *V* indicates that it is a Lyapunov function according to the endemic equilibrium ${{\widetilde{E}}}_+$ for the system (B1). Therefore, when the endemic equilibrium ${{\widetilde{E}}}_+$ exists for the system (B1), it is globally asymptotically stable with respect to the dynamics given by (B1) with the initial condition in *D*. Since the dynamics of (5) with $\epsilon = 0$ must eventually approach that of (B1), this result shows the global stability of the endemic equilibrium for (5).

## Appendix C Proof for Theorem 8.1

The characteristic equation $\det (J_{00} -\lambda E) =0$ with the Jacobi matrix $J_{00}$ according to the disease-eliminated equilibrium $E_{00}(1-\rho , 0, 0, 0)$ for the system (5) becomes

$$\begin{aligned} (-1-\lambda )(-\omega -\lambda ) \begin{vmatrix} \ \big \{1-(1-\epsilon )\rho \big \}\mathscr {R}_{00}\dfrac{\mu }{1+\mu }-(1+c) -\lambda&\big \{1-(1-\epsilon )\rho \big \}{\mathscr {R}}_{00}\dfrac{\mu }{1+\mu } \ \\ \epsilon {\mathscr {R}}_{00}\dfrac{\mu }{1+\mu }&\epsilon \mathscr {R}_{00}\dfrac{1}{1+\mu }-1 -\lambda \ \end{vmatrix} = 0. \end{aligned}$$

Hence we have two negative eigenvalues $-1$ and $-\omega$ with the other two given by the roots of the equation that the above $2\times 2$ determinant is equal to zero. Both of them have negative real parts if and only if

$$\begin{aligned} \left\{ \begin{aligned}&\big \{1-(1-\epsilon )\rho \big \}\mathscr {R}_{00}\dfrac{\mu }{1+\mu }-(1+c) + \epsilon \mathscr {R}_{00}\dfrac{1}{1+\mu }-1 <0; \\&\Big [ \big \{1-(1-\epsilon )\rho \big \}\mathscr {R}_{00}\dfrac{\mu }{1+\mu }-(1+c) \Big ] \Big ( \epsilon \mathscr {R}_{00}\dfrac{1}{1+\mu }-1 \Big ) \\&\quad \quad \quad - \big \{1-(1-\epsilon )\rho \big \}{\mathscr {R}}_{00}\dfrac{\mu }{1+\mu } \cdot \epsilon \mathscr {R}_{00}\dfrac{\mu }{1+\mu } >0, \end{aligned} \right. \end{aligned}$$

that is,

$$\begin{aligned} \left\{ \begin{aligned}&\big \{1-(1-\epsilon )\rho \big \}\mathscr {R}_{00}\dfrac{\mu }{1+\mu }-(1+c) + \epsilon \mathscr {R}_{00}\dfrac{1}{1+\mu }-1<0; \\&\big \{1-(1-\epsilon )\rho \big \}\mathscr {R}_{00}\dfrac{\mu }{1+\mu }-(1+c) + (1+c) \epsilon \mathscr {R}_{00}\dfrac{1}{1+\mu } <0. \end{aligned} \right. \end{aligned}$$

Therefore we find that the second inequality gives the necessary and sufficient condition that every eigenvalue has a negative real part. It can be expressed as the inverse inequality of (9). At the same time, we can find that, if the inverse of the above second inequality is satisfied, there exists an eigenvalue with a positive real part. Then the disease-eliminated equilibrium $E_{00}$ is unstable.

## Appendix D Proof for Corollary 8.1.1

It is sufficient to show that $\{ G(\mu , \rho )\}^{-1} < 1/(\epsilon {\mathscr {R}}_{00})$ when ${\mathscr {R}}_0 \le 1$. From the definition of ${\mathscr {R}}_0$ defined by (4), we have ${\mathscr {R}}_0 \le 1$ if and only if

$$\begin{aligned} \dfrac{1}{\epsilon {\mathscr {R}}_{00}} \ge \dfrac{\, 1\, }{\epsilon } \Big \{ 1-(1-\epsilon )\rho \dfrac{\mu }{1+\mu } \Big \}. \end{aligned}$$

Then we can derive

$$\begin{aligned}&\dfrac{\, 1\, }{\epsilon } \Big \{ 1-(1-\epsilon )\rho \dfrac{\mu }{1+\mu } \Big \} - \{ G(\mu , \rho )\}^{-1} \\&\quad = \dfrac{1-\epsilon }{\epsilon } + \dfrac{\mu }{1+\mu } \Big \{ 1-\dfrac{\, 1\, }{\epsilon }\dfrac{1}{1+c}-\rho \dfrac{1-\epsilon }{\epsilon }\big (1-\dfrac{1}{1+c}\big ) \Big \} \\&\quad > \dfrac{1-\epsilon }{\epsilon } + \dfrac{\mu }{1+\mu } \Big ( 1-\dfrac{\, 1\, }{\epsilon }-\rho \dfrac{1-\epsilon }{\epsilon } \Big ) = \dfrac{1-\epsilon }{\epsilon } \Big \{ 1-(1-\rho )\dfrac{\mu }{1+\mu } \Big \} \ge 0. \end{aligned}$$

Therefore, we find that $\{ G(\mu , \rho )\}^{-1} < 1/(\epsilon {\mathscr {R}}_{00})$ when ${\mathscr {R}}_0 \le 1$, and then the inverse equality of (9) is satisfied.

## Appendix E Proof for Theorem 8.2

First we consider the case of $\rho =1$. From (11), we can derive the following equations about $y_{\textrm{v}}^*$ and $y_{\textrm{r}}^*$:

E4 $$\begin{aligned} y_{\textrm{r}}^* = \dfrac{(1+c)y_{\textrm{v}}^*}{1+cy_{\textrm{v}}^*}; \ \varphi (y_{\textrm{v}}^*) := \dfrac{\epsilon \mathscr {R}_{00}}{1+\mu } \Big ( \dfrac{1+c}{1+cy_{\textrm{v}}^*}+\mu \Big ) (1-y_{\textrm{v}}^*) - (1+c) =0. \end{aligned}$$

The function $\varphi (y)$ is monotonically decreasing in terms of $y >0$, and $\varphi (1) = -(1+c) < 0$. Hence, if and only if $\varphi (0) >0$, the equation $\varphi (y) = 0$ has a unique positive root $y = y_{\textrm{v}}^* < 1$. We remark from the first equation of (E4) that $y_{\textrm{r}}^*\in (0, 1)$ is uniquely determined for each positive $y_{\textrm{v}}^* < 1$. Therefore, if and only if $\varphi (0) >0$, the endemic equilibrium $E_{++}$ exists when $\rho = 1$. It is easy to show that the condition that $\varphi (0) >0$ is necessary and sufficient to make (9) hold with $\rho = 1$.

Next, from (10) in the case of $\mu > 0$ and $\rho < 1$, we can derive the equation $\psi (\zeta ^*) = 0$ in terms of $\zeta ^*:= (1-\rho -x_{\textrm{v}}^*)/x_{\textrm{v}}^*$ with

E5 $$\begin{aligned} \psi (\zeta )&:= \dfrac{c(1+\mu )}{\ {\mathscr {R}}_{00}} - \dfrac{\epsilon }{1/c+\epsilon \zeta } - \dfrac{\mu }{1/c+1-\epsilon } \Big \{ \epsilon \dfrac{1/c+\rho (1-\epsilon )}{1/c+1+\epsilon \zeta }+\dfrac{(1-\epsilon )(1-\rho )}{1+\zeta } \Big \}. \end{aligned}$$

Since $x_{\textrm{v}}^*\in (0, 1-\rho )$ for the endemic equilibrium, we have $\zeta ^*\in (0, \infty )$. We can easily find that $\psi (\zeta )$ is monotonically increasing in terms of $\zeta >0$. Further $\psi (\zeta )\rightarrow c(1+\mu )/{\mathscr {R}}_{00}$ as $\zeta \rightarrow \infty$. Hence the equation $\psi (\zeta ) = 0$ necessarily has a unique positive root $\zeta ^*$ if and only if $\psi (0) < 0$, which can be easily proved to be equivalent to the condition (9). With $\zeta ^* > 0$, the equilibrium value $x_{\textrm{v}}^*\in (0, 1-\rho )$ is uniquely determined by $x_{\textrm{v}}^* = (1-\rho )/(1+\zeta ^*)$.

On the other hand, with the second equation of (10), we can derive

$$\begin{aligned} x_{\textrm{v}}^*+y_{\textrm{v}}^*&= g(x_{\textrm{v}}^*) := \dfrac{1/c}{1+1/c-\epsilon } x_{\textrm{v}}^* + \dfrac{1-\epsilon }{1+1/c-\epsilon }\Big \{\dfrac{(1-\rho )(1+1/c)}{1+1/c-\epsilon }-\dfrac{\epsilon }{1-\epsilon }\Big \} \\&\quad + \dfrac{\epsilon (1-\rho ) (1+1/c)}{(1+1/c-\epsilon )^2} \dfrac{1/c+(1-\epsilon )\rho }{(1+1/c-\epsilon )x_{\textrm{v}}^*+\epsilon (1-\rho )} . \end{aligned}$$

Then we can easily find that *g*(*x*) is concave with $g(0) = 1$ and $g(1-\rho ) = 1-\rho < 1$, so that $g(x) < 1$ for $x\in (0, 1-\rho )$. This result indicates that, if the equation $\psi (x) = 0$ has a unique positive root $x_{\textrm{v}}^*\in (0, 1-\rho )$, the value $y_{\textrm{v}}^*$ is reasonably determined by the second equation of (10) such that $x_{\textrm{v}}^*+y_{\textrm{v}}^* < 1$. Moreover, by the third equation of (10), the value $y_{\textrm{r}}^*$ is reasonably determined at the same time such that $0< y_{\textrm{r}}^* < 1$. Finally, from these arguments, if and only if $\psi (0) < 0$, which is equivalent to the condition (9), the endemic equilibrium $E_{++}$ uniquely exists when $\rho < 1$.

## Appendix F Proof for Theorem 8.3

The Jacobi matrix about the endemic equilibrium $E_{++}$ becomes

$$\begin{aligned} J( E_{++ } ) := \begin{pmatrix} - (B+c) &{} - \dfrac{\mu }{1+\mu } {\mathscr {R}}_{00} x_{\textrm{v}}^{*} &{} 0 &{} - \dfrac{1}{1+\mu } {\mathscr {R}}_{00} x_{\textrm{v}}^{*} \\ ( 1- \epsilon )B &{} \mu \Xi - (\epsilon B+1+c) &{} 0 &{} \Xi \\ 0 &{} 0 &{} - (B+\omega ) &{} 0 \\ 0 &{} \mu \Phi &{} (1 - \epsilon ) B &{} \Phi - \epsilon B - 1\ \end{pmatrix}, \end{aligned}$$

where

$$\begin{aligned}&B := {\mathscr {R}}_{00} \dfrac{y_{\textrm{r}}^{*} + \mu y_{\textrm{v}}^{*}}{ 1 + \mu }; \ \Phi := \dfrac{y_{\textrm{r}}^{*}}{y_{\textrm{r}}^{*} + \mu y_{\textrm{v}}^{*}}; \\&\Xi := \dfrac{1+c}{\mu }\dfrac{\mu y_{\textrm{v}}^{*}}{y_{\textrm{r}}^{*} + \mu y_{\textrm{v}}^{*}} = \dfrac{1+c}{\mu } \Big ( 1 - \dfrac{y_{\textrm{r}}^{*}}{y_{\textrm{r}}^{*} + \mu y_{\textrm{v}}^{*}} \Big ) = \dfrac{1+c}{\mu }(1-\Phi ). \end{aligned}$$

Then the characteristic polynomial for the eigenvalue $\lambda$ about $E_{++}$ can be obtained as $\big \vert J( E_{++ } ) - \lambda E\, \big \vert = (B+\omega +\lambda ) h(\lambda )$ with

F6 $$\begin{aligned} h(\lambda )&:= - \begin{vmatrix} - (B+c+\lambda )&0&- \dfrac{1}{1+\mu } {\mathscr {R}}_{00} x_{\textrm{v}}^{*} \\ ( 1- \epsilon )B&- (\epsilon B+1+c+\lambda )&\Xi \\ 0&\mu (\epsilon B+1+\lambda )&\Phi - \epsilon B - 1 - \lambda \ \end{vmatrix} \nonumber \\&= {\lambda }^{3} + a_{2} {\lambda }^{2} + a_{1} \lambda + a_{0}, \end{aligned}$$

where

$$\begin{aligned} a_{2}&= c\Phi + 1+c + B + 2 \epsilon B; \\ a_{1}&= (c+B+\epsilon B)c\Phi +(B+c)(2\epsilon B+1)+\epsilon B(\epsilon B+1) + \dfrac{(1- \epsilon )\mu }{1+\mu } {\mathscr {R}}_{00} x_{\textrm{v}}^{*} B; \\ a_{0}&= \epsilon B (B+c) ( \epsilon B + 1 + c\Phi ) +\dfrac{(1- \epsilon ) \mu }{1+\mu } {\mathscr {R}}_{00} x_{\textrm{v}}^{*} B (\epsilon B+1). \end{aligned}$$

Thus we have a negative eigenvalue $\lambda = -(B+\omega )$ and the cubic equation $h(\lambda ) = 0$ given by (F6) to determine the other three eigenvalues for $E_{++}$.

Every coefficient of $h(\lambda )$ is positive: $a_{2} > 0$, $a_{1}>0$, and $a_{0}>0$. Since $0< \Phi < 1$, we have

$$\begin{aligned} a_{2}&> a_2' := 1+c + B + 2 \epsilon B>0; \\ a_{1}&> a_1' := (B+c)(2\epsilon B+1)+\epsilon B(\epsilon B+1) + \dfrac{(1- \epsilon )\mu }{1+\mu } {\mathscr {R}}_{00} x_{\textrm{v}}^{*} B >0; \\ a_{0}&< a_0' := \epsilon B (B+c) (\epsilon B + 1+c) +\dfrac{(1- \epsilon ) \mu }{1+\mu } {\mathscr {R}}_{00} x_{\textrm{v}}^{*} B (\epsilon B+c), \end{aligned}$$

and subsequently find that

F7 $$\begin{aligned} a_{2} a_{1} - a_{0}> a_{2}' a_{1}' - a_{0}'&= (\epsilon B+1+c)(\epsilon B+1)(c+B+\epsilon B) \nonumber \\&\quad + (1+\epsilon )B \big \{ (B+c)(2\epsilon B+1)+\epsilon B(\epsilon B+1) \big \} \nonumber \\&\quad + \dfrac{( 1 - \epsilon ) \mu }{1+\mu } \big \{ 1 + (1+\epsilon ) B \big \} {\mathscr {R}}_{00} x_{\textrm{v}}^{*} B >0. \end{aligned}$$

Consequently from the Routh-Hurwitz criterion, we can find that all roots of $h(\lambda ) = 0$ have negative real parts. Therefore it has been proved that every eigenvalue for $E_{++}$ has negative real part. This result shows Theorem 8.3 about the local stability of $E_{++}$ when it exists.

## Appendix G Proof for Theorem 9.4 and Corollary 9.4.3

As shown in the proof for Theorem 8.2 (Appendix E), the endemic size can be determined by the unique positive root $\zeta =\zeta ^*$ of the equation $\psi (\zeta ) =0$ with (E5) for $\rho < 1$ under the condition (9). From the equation $\psi (\zeta ^*) =0$, we can derive

G8 $$\begin{aligned} \dfrac{\partial \zeta ^*}{\partial \mu } = \dfrac{1+\epsilon c\zeta ^*-\epsilon {\mathscr {R}}_{00}}{K\mu \mathscr {R}_{00}(1/c+\epsilon \zeta ^*)} \end{aligned}$$

with

$$\begin{aligned} K := \dfrac{\epsilon ^2}{(1/c+\epsilon \zeta ^*)^2} + \dfrac{\mu }{1/c+1-\epsilon } \Big \{ \epsilon ^2\dfrac{1/c+ (1-\epsilon )\rho }{(1/c+1+\epsilon \zeta ^*)^2} + \dfrac{(1-\epsilon )(1-\rho )}{(1+\zeta ^*)^2} \Big \} >0. \end{aligned}$$

First for $\epsilon {\mathscr {R}}_{00} \le 1$, we find from (G8) that $\partial \zeta ^*/\partial \mu >0$ for any $\zeta ^* > 0$ and $\mu > 0$. Since $x_{\textrm{v}}^* = (1-\rho )/(1+\zeta ^*)$, we then have $\partial x_{\textrm{v}}^*/\partial \mu < 0$. Lastly we can get the following result:

### Lemma G.1

$x_{\textrm{v}}^*$ is monotonically decreasing in terms of $\mu > 0$ when $\rho < 1$ and $\epsilon {\mathscr {R}}_{00} \le 1$.

Next for $\epsilon {\mathscr {R}}_{00} > 1$, the partial derivative (G8) indicates that $\partial \zeta ^*/\partial \mu >0$ if and only if $\zeta ^* > \zeta _c:=(\epsilon {\mathscr {R}}_{00} - 1)/(\epsilon c)$, while $\partial \zeta ^*/\partial \mu < 0$ if and only if $\zeta ^* < \zeta _c$. Since $\psi (\zeta )$ is monotonically increasing in terms of $\zeta > 0$, such that $\psi (\zeta ) < 0$ for $\zeta < \zeta ^*$ and $\psi (\zeta ) > 0$ for $\zeta > \zeta ^*$ under the condition (9), we find that $\partial \zeta ^*/\partial \mu >0$ if and only if $\psi (\zeta _c) < 0$, while $\partial \zeta ^*/\partial \mu < 0$ if and only if $\psi (\zeta _c) > 0$. Since $\zeta _c$ is independent of $\mu$, this result indicates that the sign of $\partial \zeta ^*/\partial \mu$ is determined independently of $\mu$. Hence we obtain the following lemma:

### Lemma G.2

$x_{\textrm{v}}^* > 0$ is monotonic in terms of $\mu > 0$ when $\rho < 1$.

Now we can derive

G9 $$\begin{aligned} \psi (\zeta _c) = \dfrac{\mu \{ \epsilon c -1+\epsilon ^2\mathscr {R}_{00}+\epsilon (1-\epsilon ){\mathscr {R}}_{00}\rho \} }{ \epsilon {\mathscr {R}}_{00}(1+\epsilon {\mathscr {R}}_{00}/c) \{ 1+(\epsilon {\mathscr {R}}_{00}-1)/(\epsilon c) \} }. \end{aligned}$$

Thus we can result that $\psi (\zeta _c) < 0$ if and only if $\rho < \rho _c$ where $\rho _c$ is defined by (19). That is, $\partial \zeta ^*/\partial \mu >0$ if and only if $\rho < \rho _c$. Lastly we have

### Lemma G.3

$x_{\textrm{v}}^* > 0$ is monotonically decreasing in terms of $\mu > 0$ if and only if $\rho < \rho _c$ when $\rho < 1$ and $\epsilon {\mathscr {R}}_{00} > 1$.

It can be easily found that $\rho _c < 1$ when $\epsilon {\mathscr {R}}_{00} > 1$.

In the critical case of $\rho = \rho _c > 0$ with $\epsilon \mathscr {R}_{00} > 1$, we have $\psi (\zeta _c ) = 0$ in (G9), which means that $\zeta ^* = \zeta _c$ and subsequently $\partial \zeta ^*/\partial \mu = 0$. Actually, from $\zeta ^* = \zeta _c$, we find that $x_{\textrm{v}}^* = (1-\rho )/(1+\zeta _c)$, the endemic sizes $y_{\textrm{r}}^*$, $y_{\textrm{v}}^*$, and $z^*$ defined by (18) are independent of $\mu$, as given by (20) which are derived by (10). This result gives Corollary 9.4.3.

On the other hand, from (10), we can easily find that the endemic sizes $y_{\textrm{r}}^*$, $y_{\textrm{v}}^*$, and $z^*$ are monotonically decreasing in terms of $x_{\textrm{v}}^* > 0$. Therefore, from Lemma G.2, we can get the following lemma:

### Lemma G.4

The endemic sizes $y_{\textrm{r}}^*$, $y_{\textrm{v}}^*$, and $z^*$ are monotonic in terms of $\mu$ when $\rho < 1$.

Consequently, from Lemmas G.1, G.2, G.3, and G.4, we can obtain the result of Theorem 9.4 for $\rho < 1$.

## Appendix H Proof for Corollary 9.4.1

In case of $\rho = 1$, the unique positive root of $\varphi (y) = 0$ with (E4) gives the endemic size $y_{\textrm{v}}^*$ under the condition (9) with $\rho = 1$ (Appendix E). Then, from the equation $\varphi ( y_{\textrm{v}}^*) = 0$, we can derive

H10 $$\begin{aligned} \dfrac{\partial y_{\textrm{v}}^*}{\partial \mu } = \dfrac{1-y_{\textrm{v}}^*-(1+c)/(\epsilon {\mathscr {R}}_{00})}{(1+c)^2/(1+cy_{\textrm{v}}^*)^2+\mu }. \end{aligned}$$

When $\epsilon {\mathscr {R}}_{00} \le 1+c$, we have $\partial y_{\textrm{v}}^*/\partial \mu < 0$ for any $\mu > 0$. Therefore we obtain the following result:

### Lemma H.1

The endemic sizes $y_{\textrm{r}}^*$ and $y_{\textrm{v}}^*$ are monotonically decreasing in terms of $\mu$ when $\rho = 1$ and $\epsilon {\mathscr {R}}_{00} \le 1+c$.

From the first equation in (E4), we note that the endemic size $y_{\textrm{r}}^*$ is monotonically increasing in terms of $y_{\textrm{v}}^*$.

When $\epsilon {\mathscr {R}}_{00} > 1+c$, $\partial y_{\textrm{v}}^*/\partial \mu >0$ if and only if $y_{\textrm{v}}^*< y_{\textrm{v}}^c:=1-(1+c)/(\epsilon {\mathscr {R}}_{00})$, while $\partial y_{\textrm{v}}^*/\partial \mu < 0$ if and only if $y_{\textrm{v}}^* > y_{\textrm{v}}^c$. Since $\varphi (y)$ is monotonically decreasing in terms of $y > 0$, such that $\varphi (y) > 0$ for $y < y_{\textrm{v}}^*$ and $\varphi (y ) < 0$ for $y > y_{\textrm{v}}^*$. Hence, $\partial y_{\textrm{v}}^*/\partial \mu >0$ if and only if $\varphi (y_{\textrm{v}}^c)<0$, while $\partial y_{\textrm{v}}^*/\partial \mu < 0$ if and only if $\varphi (y_{\textrm{v}}^c)>0$. Now we can derive

$$\begin{aligned} \varphi (y_{\textrm{v}}^c) =\dfrac{c(1+c)}{(1+\mu )(\epsilon {\mathscr {R}}_{00}-c)} > 0 \end{aligned}$$

for $\epsilon {\mathscr {R}}_{00} > 1+c$. Therefore we have the following result:

### Lemma H.2

The endemic sizes $y_{\textrm{r}}^*$ and $y_{\textrm{v}}^*$ are monotonically decreasing in terms of $\mu$ when $\rho = 1$ and $\epsilon {\mathscr {R}}_{00} > 1+c$.

Consequently, from Lemmas H.1 and H.2, the endemic sizes $y_{\textrm{r}}^*$ and $y_{\textrm{v}}^*$ are monotonically decreasing in terms of $\mu$ when $\rho = 1$.
